# Oxidation of benzylic alcohols to carbonyls using *N*-heterocyclic stabilized λ^3^-iodanes

**DOI:** 10.3762/bjoc.20.149

**Published:** 2024-07-19

**Authors:** Thomas J Kuczmera, Pim Puylaert, Boris J Nachtsheim

**Affiliations:** 1 Institute for Organic and Analytical Chemistry, University of Bremen, Bremen, Germany; 2 Institute for Inorganic Chemistry and Crystallography, University of Bremen, Bremen, Germany

**Keywords:** alcohol oxidation, hypervalent iodine, *N*-heterocycles

## Abstract

We present *N*-heterocycle-stabilized iodanes (NHIs) as suitable reagents for the mild oxidation of activated alcohols. Two different protocols, both involving activation by chloride additives, were used to synthesize benzylic ketones and aldehydes without overoxidation in up to 97% yield. Based on MS experiments an activated hydroxy(chloro)iodane is proposed as the reactive intermediate.

## Introduction

**Figure 1:**
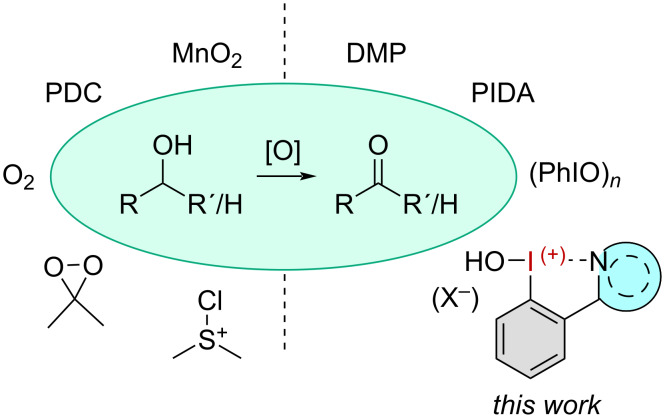
Overview of common non-iodine-based (left) and iodine-based (right) oxidizing reagents for the generation of carbonyls from the corresponding alcohols.

The oxidation of alcohols to aldehydes and ketones is an essential transformation in organic chemistry [1–2]. Generating aldehydes is particularly challenging as they are easily overoxidized to carboxylic acids. Over the past decades a variety of methods have been developed, utilizing toxic heavy metals such as pyridinium dichromate (PDC) [3–5] or manganese dioxide (Figure 1) [6–7]. Molecular oxygen [8] and peroxides [9–10] can also be used as inexpensive terminal oxidants in combination with transition-metal catalysts. Metal-free methods employ chlorodimethylsulfonium compounds as the reactive species and have gained great popularity under the name Swern oxidation or the Corey–Kim oxidation [11]. Hypervalent iodine compounds have also been studied and are well established in several oxidative transformations including the synthesis of complex molecules and drugs [12–13]. The most prominent examples are the pentavalent derivatives 2-iodoxybenzoic acid (IBX) and Dess–Martin periodinane (DMP) [14–15]. Although mild and selective oxidants, these highly oxidized λ^5^-iodanes have drawbacks, in particular low solubility and moisture sensitivity [11]. Hypervalent iodine compounds in a lower oxidation state (λ^3^-iodanes), such as iodosobenzene (PhIO)*_n_* or phenyliodine(III) diacetate (PIDA) have been reported in alcohol oxidations but they often result in overoxidation to the corresponding carboxylic acids [16]. Additives such as bromide salts or Al_2_O_3_ can eliminate this problem and allow selective oxidation to some extent [17–20].

During the past years, *N*-heterocycle-stabilized iodanes (NHIs) were demonstrated as suitable tools for various applications among them group transfer reactions [21] and as building blocks [22–24]. The synthetic potential of NHIs has been previously studied in model transformations such as thioanisole oxygenation, oxidative lactonization, or diacetoxylation of alkenes [25–28]. In this work, we want to apply NHIs in a mild oxidation of primary and secondary benzylic alcohols to aldehydes and ketones as an alternative to λ^5^-iodanes.

## Results and Discussion

**Figure 2:**
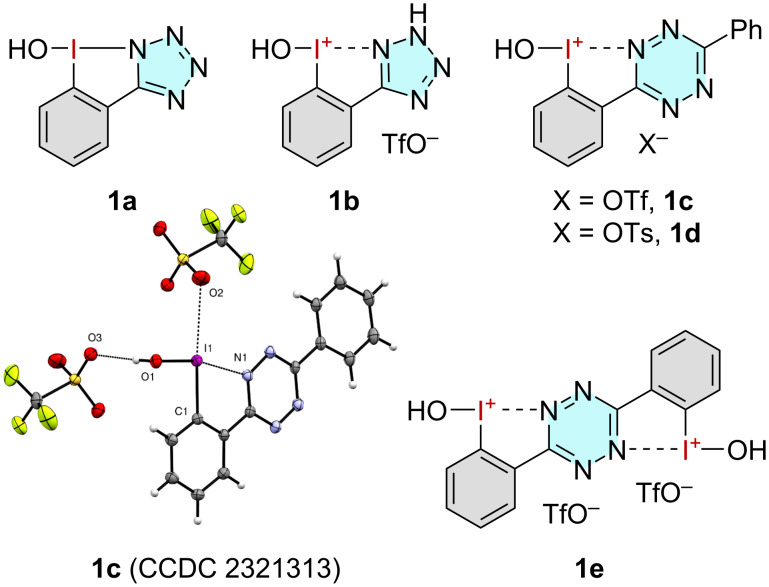
NHIs investigated for the oxidation of benzylic alcohols and the crystal structure (ORTEP drawing) of **1c** (CCDC 2321313), showing the coordination of the triflate to two positions of the iodane. Thermal ellipsoids are displayed with 50% probability. Selected bond lengths and angles: I1–N1: 2.44(4) Å; I1–O1: 1.94(9) Å; I1–O2: 3.04(1) Å; C1–I1–N1: 73.5(8)°; O1–I1–N1: 166.6(5)°; N1–I1–C1–O1: 177.8(3)°.

Initially, we investigated a variety of pyrazole-, triazole-, and oxazole-substituted hydroxy-NHIs previously developed by our group [25]. However, none of them proved to be effective in a model oxidation reaction of *n*-octanol (**2**). Since previous investigations have repeatedly shown that the number of heteroatoms in the *N*-heterocycle correlates with the NHIs activity, a series of tetrazole- and tetrazine-substituted NHIs **1a**–**e** was synthesized (Figure 2) [29–30]. A crystal structure was additionally obtained for tetrazine **1c**. Bond lengths and angles were similar to those of known five-membered NHIs [25], including a strong intramolecular interaction between the nitrogen of the tetrazine and the hypervalent iodine atom (I1–N1: 2.44(4) Å; the sum of VdW radii: 3.61 Å [31]).

**Figure 3:**
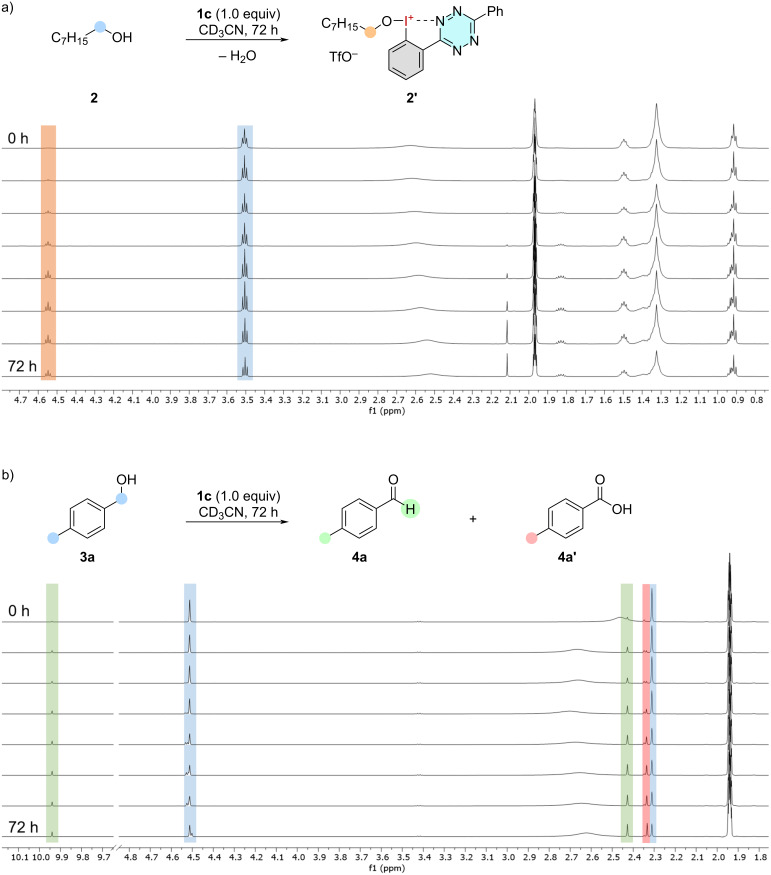
^1^H NMR spectra of the time-dependent formation of a) an alkoxy-NHI which is causing a significant downfield shift of the protons in *alpha*-position (orange) compared to the free alcohol **2** (blue) and b) oxidation of *p*-tolylmethanol (**3a**, blue) to the aldehyde **4a** (green) and carboxylic acid **4a’** (red). Reaction conditions: An equimolar mixture of NHI **1c** (10.0 µmol) and alcohol (**2** or **3a**, 10.0 µmol) were dissolved in CD_3_CN (600 µL) and ^1^H NMR spectra were recorded.

Beginning with the electron-deficient and thereby highly reactive NHIs **1a** and **1c**, we explored the potential for a ligand-exchange process on the iodane via ^1^H NMR spectroscopy by combining equimolar quantities of NHI and *n*-octanol (**2**). When the tetrazole-substituted hydroxy(aryl)iodane **1a** was added, no significant shifts in the NMR spectral signals were detected, probably due to the poor solubility of the iodane. Conversely, with the addition of the red tetrazine salt **1c**, a significant downfield shift was observed for the *alpha*-carbon protons from 3.51 ppm to 4.55 ppm, as illustrated in Figure 3a.

This indicates a ligand exchange of the hydroxy group resulting in a loss of electron density and the formation of the alkoxy-NHI **2'**. The chemical shift is consistent with previously measured alkoxyiodanes [32].

**Figure 4:**
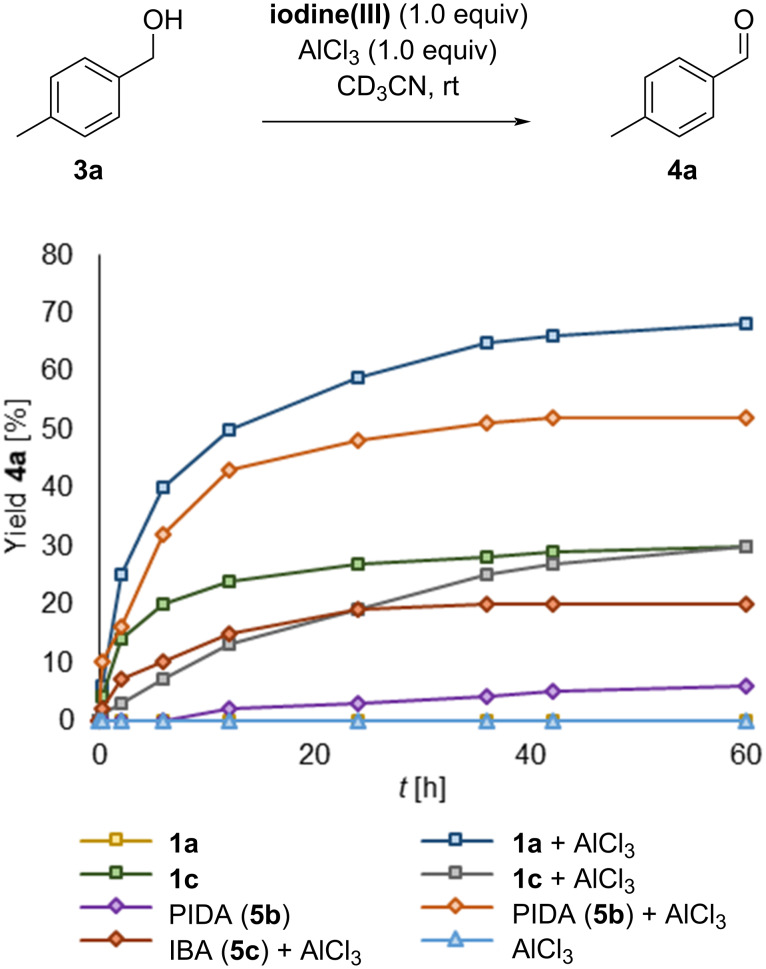
Oxidation of **3a** to **4a** using different iodine(III) reagents with AlCl_3_ as an additive. Conditions: The turnover of an equimolar mixture of **3a**, iodine(III), and AlCl_3_ (10.0 µmol, respectively) in CD_3_CN (500 µL) was monitored via ^1^H NMR spectroscopy.

The experiments were repeated using activated *p*-tolylmethanol (**3a**), again showing no reaction with iodane **1a**. Utilizing the tetrazine **1c**, *p*-methylbenzaldehyde (**4a**) was observed as a new species at 9.94 ppm (Figure 3b). The reaction reached 31% conversion after 72 h, however, *p*-methylbenzoic acid (**4a’**) was formed in 35% as well, showing an undesired overreaction. In this experiment no formation of an alkoxyiodane was observed, indicating that the formation of this ligand-exchanged intermediate is slower than the dehydrogenation. As a consequence, we attempted to accelerate the ligand exchange through the addition of a Lewis acid and the performance of the NHIs was compared with common iodine(III) reagents by ^1^H NMR spectroscopy (Figure 4). After 60 h the measurements revealed a higher yield of aldehyde **4a** using **1a** (68%) compared to **1c** (30%) under the influence of AlCl_3_. As a comparison, the use of PIDA (**5b**) and IBA (**5c**) with the additive resulted in a significantly lower oxidation of the alcohol. Only small amounts of benzoic acid **4a’** were observed in all reactions with additional AlCl_3_, suggesting that the additive inhibits the previously observed overoxidation.

Surprisingly AlCl_3_ activated the cyclic tetrazole iodane **1a** but had almost no influence on the reactivity of the tetrazine salt **1c**. Based on these results, the reaction conditions were further optimized using NHI **1a** with the benzyl alcohols **3a** (electron-rich) and **3b** (electron-poor) as the model substrates. First, the reaction temperature was increased, finding 60 °C to be the optimal value in EtOAc (Table 1, entry 1). At this temperature, the reaction time was significantly reduced to 2.5 h. A variety of other additives were tested next, revealing TsOH or NaOTs inhibiting the reaction (Table 1, entries 2 and 3). The addition of tetrabutylammonium halides showed the chloride salt being superior, giving comparable or even better yields than AlCl_3_ (Table 1, entries 4–7). Investigation of other chloride sources resulted in a reduced yield in the case of ammonium chloride and an improved yield of 82% of **4a** when concentrated aqueous HCl was added (Table 1, entries 8 and 9). Other solvents did not further increase the yield (see the full table in Supporting Information File 1).

**Table 1:** Varying the additive and solvent in the oxidation of electron-rich and electron-deficient benzylic alcohols with **1a**.^a^

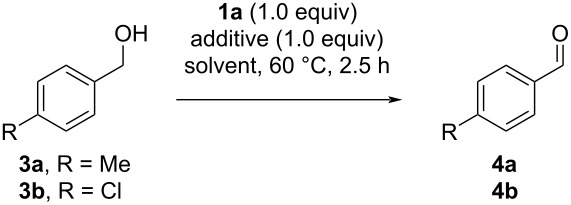				

Entry	Additive	Solvent	Yield [%]	

			**4a**	**4b**

1	AlCl_3_	EtOAc	65	39
2	TsOH∙H_2_O	EtOAc	1	1
3	NaOTs	EtOAc	1	1
4	TBAF	EtOAc	9	19
5	TBACl	EtOAc	67	62
6	TBABr	EtOAc	58	47
7	TBAI	EtOAc	40	36
8	NH_4_Cl	EtOAc	37	26
9	HCl	EtOAc	82	44
10	TBACl	MeCN	64	69
11^b^	TBACl	MeCN	74	78
12^b^	HCl	EtOAc	90	53

^a^Reaction conditions: **1a** (100 µmol), **3a**/**3b** (100 µmol), and the additive (100 µmol) were stirred in the given solvent (1 mL) at 60 °C for 2.5 h and quenched with Me_2_S (200 µmol). ^b^Optimum reaction conditions were used: **1a** (100 µmol), **3a**/**3b** (100 µmol), and the additive (100 µmol) were stirred in the given solvent (0.5 mL) at 60 °C for 2.5 h and quenched with Me_2_S (200 µmol). The yield was determined via ^1^H NMR using tetraethylsilane as an internal standard.

However, when electron-deficient *p*-chlorobenzyl alcohol (**3b**) was used the highest yield of **4b** (69%) was achieved with TBACl as the chloride source in MeCN (Table 1, entry 10). These optimizations lead to the conclusion that AlCl_3_, as proposed in the initial experiments is not a Lewis acid activator but just a chloride source. Further optimization studies improved the yield to 78% of **4b** using a concentration of 0.20 M of the alcohol and 1.4 equiv of **1a** (see Supporting Information File 1). Finally, all NHIs were tested under the optimized conditions, revealing the tetrazole-substituted iodane **1a** to be the best oxidant for this reaction (Table 2).

**Table 2:** Testing different NHIs under the optimum conditions for oxidation of electron-deficient substrate **3b**.^a^

Iodane	Yield of **4b** [%]

**1a**	78
**1b**	71
**1c**	46
**1d**	29
**1e**	41

^a^Reaction conditions: NHI (**1a**–**d**: 140 µmol, **1e**: 70.0 µmol), *p*-chlorobenzyl alcohol (**3b**, 100 µmol) and TBACl (100 µmol) in MeCN (500 µL) were stirred at 60 °C for 2.5 h and quenched with Me_2_S (200 µmol). The yield was determined via ^1^H NMR with tetraethylsilane as an internal standard.

**Figure 5:**
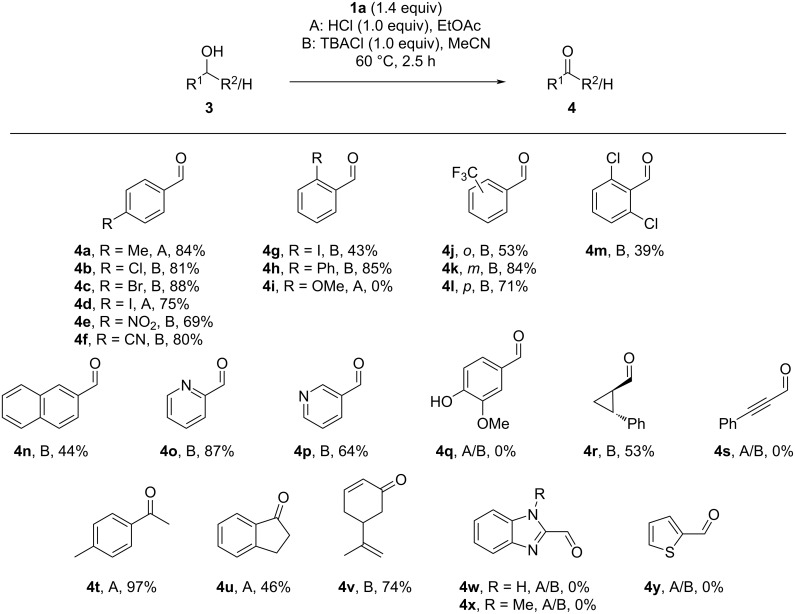
Substrate scope of aldehydes and ketones synthesized from the corresponding alcohols. Isolated yields. Reaction conditions: **1a** (700 µmol), alcohol (500 µmol), and method A HCl (37%, 500 µmol) in EtOAc (2.5 mL) or method B TBACl (500 µmol) in MeCN (2.5 mL), respectively, were stirred at 60 °C for 2.5 h and quenched with Me_2_S (1.40 mmol).

The two suitable methods (A: HCl in EtOAc; B: TBACl in MeCN) were then applied to a variety of activated alcohols. The best option is shown in Figure 5. Model substrate **4a** could be isolated in a high yield of 84% with reisolation of the 5-(2-iodophenyl)-1*H*-tetrazole (**6**) in 90% yield. Other *para*-halogenated benzaldehydes **4b**–**f** were isolated in good yields of up to 88%. *ortho*-Substitution led to a lower yield of the iodinated product **4g** (43%) compared to the *para*-iodinated analogues **4d** (75%). The *ortho*-phenyl-substituted aldehyde **4h** was isolated in 85% yield, while the *ortho*-methoxy substrate did not convert to **4i**. The *ortho*-, *meta*- and *para*-permutation of a CF_3_ group showed lower reactivity for the *ortho*-substituted **4j** (53%), while the *meta*- and *para*-derivatives **4k** and **4l** gave higher yields of 84% and 71%, respectively. The steric inhibition of a doubly substituted phenyl ring was observed in a diminished formation of 2,6-dichlorobenzaldehyde (**4m**) in 39% yield. Naphthalen-2-ylmethanol gave aldehyde **4n** in 44% yield. Pyridines **4o** and **4p** were also compatible and gave good yields of 87% and 64%, respectively. Unfortunately, the synthesis of vanillin (**4q**) was unsuccessful due to undesirable oxidation reactions of the electron-rich arene. The cyclopropane derivative **4r** was generated from the cyclopropylmethanol in 53% yield. The acetylene derivative **4s** could not be isolated due to undesired oxidations of the triple bond. The behavior of secondary benzylic alcohols was tested next, giving 4-methylacetophenone (**4t**) in an excellent yield of 97% and 1-indanone (**4u**) in 46%. It is worth noting that for some derivatives oxidized by method A, an acylation of the alcohol was detected as a side reaction via mass spectrometry. Vinyl alcohols were also studied, giving carvone (**4v**) in 74% yield without oxidation of the double bonds. Finally, other heterocyclic benzylic alcohols were investigated, which led to undesired chlorinations in the case of benzimidazoles **3w** and **3x** and decomposition for thiophenylmethanol **3y**.

**Scheme 1:**
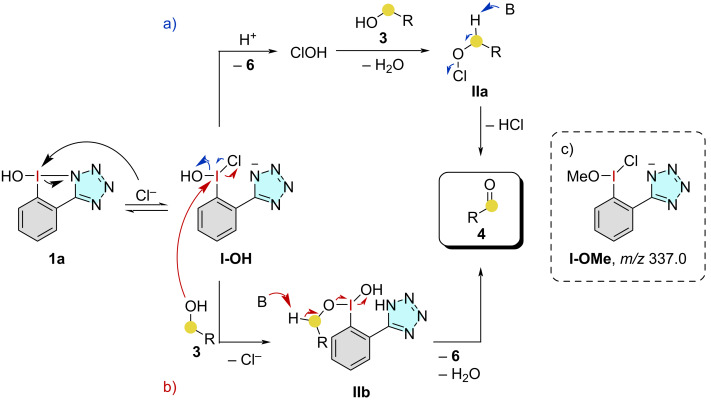
Possible reaction mechanisms via the formation of a) a Cl(I) species and b) the formation of an alkoxyiodane **IIb**. Both are initialized by the activated iodane **I-OH**, which was observed as c) **I-OMe** species in the ESI(−) MS.

Regarding the reaction mechanism, two plausible pathways can be discussed based on literature examples (Scheme 1, path a [17] and path b [33]). In either path, initial ligand exchange to the hydroxy(chloro)iodane **I-OH** is proposed. For getting an indication of a chloride-activated iodane of this type, a mixture of NHI **1a** and HCl in EtOAc was stirred for 1 h at 60 °C and an ESI(−) mass spectrum was recorded afterward, showing an ion **I-OMe** with *m/z* 337.0 [**1a** – OH + MeO + Cl]^−^ (Scheme 1c). It is known that methanol, which is used as a solvent in the mass spectrometer, can be exchanged with the hydroxy group of the NHI [21]. No such ion was measured in the mixture before heating. This ion therefore indicates an I–Cl bond in the activated iodane. Starting from **I**-**OH**, in a potential path a) formation of hypochlorous acid is suggested, which consequently oxidizes the alcohol through the alkyl hypochlorite **IIa**. The second mechanism (path b) requires a direct ligand exchange of **I-OH** with the alcohol and subsequent β-elimination of the alkoxy(hydroxy)iodane **IIb** to form the desired aldehyde **4**.

## Conclusion

In conclusion, this study has successfully introduced *N*-heterocycle-stabilized iodanes (NHIs) as effective λ^3^-iodane oxidants for the selective synthesis of ketones and aldehydes, avoiding overoxidation to carboxylic acids. The developed protocols proved particularly effective for benzylic alcohols, yielding good to excellent results. The beneficial role of chloride salt additives was investigated, potentially leading to the formation of a hydroxy(chloro)iodane intermediate. This intermediate either liberates hypochlorous acid as the terminal oxidant or undergoes a direct ligand exchange with the alcohol, followed by oxidative elimination to form the aldehyde. Thus, these reagents offer a viable alternative to traditional aryl-λ^5^-iodane-based oxidants, although further studies are necessary to fully understand their reaction mechanisms.

## Experimental

### General procedure for oxidation of benzylic alcohols

**1a** (700 µmol, 201 mg, 1.40 equiv), benzylic alcohol (**3**, 500 µmol, 1.00 equiv) and method A: aqueous HCl (37%, 500 µmol, 41.6 µL, 1.00 equiv) in EtOAc (2.5 mL) or method B: TBACl (500 µmol, 137 mg, 1.00 equiv) in MeCN (2.5 mL), respectively, were stirred at 60 °C for 2.5 h, quenched with Me_2_S (2.00 equiv) and the reaction mixture was purified via flash column chromatography on silica.

## Supporting Information

File 1 Experimental part and copies of spectra.

## Data Availability

All data that supports the findings of this study is available in the published article and/or the supporting information to this article
