# Supplementary material for: Oxidation of benzylic alcohols to carbonyls using N-heterocyclic stabilized λ3-iodanes
Source: Beilstein J Org Chem. 2024 Jul 19;20:1677–83. doi: 10.3762/bjoc.20.149 (PMC11285054; doi:10.3762/bjoc.20.149)
Supplement: File 1 — Experimental part and copies of spectra. [file Beilstein_J_Org_Chem-20-1677-s001.pdf]

BEILSTEIN JOURNAL OF ORGANIC CHEMISTRY

Supporting Information

for

Oxidation of benzylic alcohols to carbonyls using *N*-heterocyclic stabilized λ^3-iodanes

Thomas J. Kuczmera, Pim Puylaert and Boris J. Nachtsheim

Beilstein J. Org. Chem. **2024,** *20,* 1677–1683. doi:10.3762/bjoc.20.149

Experimental part and copies of spectra

License and Terms: This is a supporting information file under the terms of the Creative Commons Attribution License (https://creativecommons.org/licenses/by/4.0). Please note that the reuse, redistribution and reproduction in particular requires that the author(s) and source are credited and that individual graphics may be subject to special legal provisions.
The license is subject to the *Beilstein Journal of Organic Chemistry* terms and conditions: (https://www.beilstein-journals.org/bjoc/terms)

Table of contents

1 General information S2

2 Synthesis of iodanes S3

3 Optimization data S8

4 Oxidation of benzylic alcohols S13

4.1 General procedure for oxidation of electron rich alcohols (GP A) S13

4.2 General procedure for oxidation of electron poor alcohols (GP B) S13

4.3 Substrate scope S13

5 Mechanistic studies S19

6 References S21

7 NMR spectra S22

8 Crystal structure of **1c** S32

1 General information

Unless otherwise stated, all reactions with moisture- or oxygen-sensitive reagents were performed using standard Schlenk techniques under a nitrogen or argon atmosphere. Reagents were used as received from their commercial supplier (abcr, Acros Organics, Alfa Aesar, Apollo Scientific, Carbolution Chemicals, Sigma Aldrich, TCI, fluorochem, BLD pharm). *m*CPBA was dried under vacuum (10^{-3} mbar) for 2 h before use. Anhydrous dichloromethane (DCM), acetonitrile (MeCN), tetrahydrofuran (THF) and toluene were obtained from an *inert* PS-MD-6 solvent purification system. All other solvents were dried using standard methods. [1] Unless otherwise stated, all yields refer to isolated yields of compounds estimated to be >95% pure as determined by ^{1}H NMR spectroscopy.

Thin layer chromatography was performed on fluorescence indicator marked precoated silica gel 60 plates (Macherey-Nagel, ALUGRAM Xtra SIL G/UV_{254}) and visualized by UV light (254 nm/366 nm). Flash column chromatography was performed on silica gel (0.040–0.063 mm) with the solvents given in the procedures.

^{1}H, ^{13}C, and ^{19}F spectra were recorded on Bruker Avance Neo 600-spectrometers. Chemical shifts for ^{1}H NMR spectra were reported as δ (parts per million) relative to the residual proton signal in $CDCl_3$ at 7.26 ppm (s), d_4-MeOH at 3.31 ppm (quin.), d_6-DMSO at 2.50 ppm (quin) or d_3-MeCN at 1.94 ppm (quin). Chemical shifts for ^{13}C NMR spectra were reported as δ (parts per million) relative to the signal of $CDCl_3$ at 77.0 ppm (t), CD_3OD at 49.0 ppm (sept.), d_6-DMSO at 39.5 ppm (sept.) or CD_3CN at 118.26 ppm (s). ^{19}F NMR spectra were reported as δ (parts per million) relative to $CFCl_3$ at 0.00 ppm as external standard. The following abbreviations were used to describe splitting patterns: br = broad, s = singlet, d = doublet, t = triplet, q = quartet, quin = quintet, sext. = sextet, sept = septet, m = multiplet. Coupling constants *J* are given in hertz.

HR-ESI mass spectra were recorded on a Bruker impact II. APCI mass spectra were recorded on an Advion Expression CMS^{L} via ASAP probe or direct inlet. EI mass spectra were obtained from an Agilent 7890B GC System with an Agilent 5977A MSD mass spectrometer. All signals were reported with the quotient from mass to charge *m*/*z*. Many iodonium salts undergo reductive ring-opening reactions during HRMS measurement.

IR spectra were recorded on a Nicolet Thermo iS10 scientific spectrometer with a diamond ATR unit. The absorption bands were reported in cm^{-1}.

Melting points were determined on a Büchi M-5600 melting point apparatus with a heating rate of 5 °C/min. The melting points were reported in °C. Most of the hypervalent iodine compounds underwent changes in appearance (e.g. softening) before final melting/decomposition.

Single crystals were grown from MeCN solution. A suitable crystal was selected and measured on a Bruker D8 Venture diffractometer. The crystal was kept at 100 K during data collection. Using Olex2 [2], the structure was solved with the ShelXT [3] structure solution program using intrinsic phasing and refined with the XL [4] refinement package using least squares minimization. The ORTEP drawing was made using the program Mercury from the CCDC.

2 Synthesis of iodanes

5-(2-Iodophenyl)-1*H*-tetrazole (6)

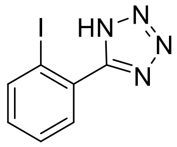

Following a literature known procedure [5] 2-iodobenzonitrile (4.58 g, 20.0 mmol), NH_4Cl (2.14 g, 40.0 mmol) and NaN_3 (2.60 mmol, 40.0 mmol) were stirred in DMF (100 mL) at 130 °C for 24 h. Water (300 mL) was added and afterwards acidified with aq. HCl (37%) to pH 1–2. The solution was stored at 4 °C for 18 h, the formed yellowish crystals were filtered, washed with water (3 × 50 mL) and dried in vacuum to obtain **6** (3.92 g, 14.4 mmol, 72%).

^{1}H NMR (600 MHz, DMSO-d_6) δ (ppm) 8.09 (d, *J* = 7.9 Hz, 1H), 7.62 – 7.57 (m, 2H), 7.35 (ddd, *J* = 8.0, 5.7, 3.5 Hz, 1H). **^{13}C NMR** (151 MHz, DMSO-d_6) δ (ppm) 156.3, 139.8, 132.4, 131.2, 130.5, 128.5, 97.6. **MS** (ESI+) *m*/*z* 272.9 $[M+H]^+$. **IR** (ATR) ν (cm^{-1}) 2446, 1823, 1599, 1471, 1438, 1390, 1163, 1051, 989, 930, 770, 744, 714. **M$_P$**: 222-223 °C. The data is in accordance with the literature. [5]

3,6-Diphenyl-1,2,4,5-tetrazine (S1)

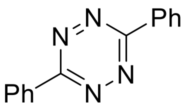

Following a literature known procedure [6] to a suspension of $Zn(OTf)_2$ (505 mg, 1.50 mmol) in benzonitrile (3.09 mL, 30.0 mmol) under an Ar atmosphere was added hydrazine hydrate (7.2 mL, 150 mmol) and the mixture was stirred at 90 °C for 4 h. The reaction was cooled to 0 °C, added to a solution of sodium nitrate (90 mL, 1.0 M) and HCl (2.0 M) was added dropwise until pH 3 was achieved. The mixture was extracted with dichloromethane (4 × 300 mL), the combined organic layers were dried over Na_2SO_4, filtered and the solvent was removed under reduced pressure. The residue was purified via column chromatography on silica (Cy 98:2 EtOAc) to obtain 3,6-diphenyl-1,2,4,5-tetrazine (**S1**, 3.29 g, 14.0 mmol, 47%) as a purple solid.

^{1}H NMR (600 MHz, $CDCl_3$) δ (ppm) 8.66 (dd, *J* = 8.0, 1.7 Hz, 4H), 7.91 – 7.49 (m, 6H). **^{13}C NMR** (151 MHz, $CDCl_3$) δ (ppm) 164.1, 132.8, 131.9, 129.5, 128.1. **MS** (ESI+) *m*/*z*

235.0 [M+H]$^+$. **IR** (ATR) ν (cm^{-1}) 3071, 3016, 2970, 1738, 1374, 1217, 917, 765, 685. **M$_P$**: 189-190 °C. The data is in accordance with the literature. [6]

3-(2-Iodophenyl)-6-phenyl-1,2,4,5-tetrazine (S2a) and 3,6-bis(2-iodophenyl)-1,2,4,5-tetrazine (S2b)

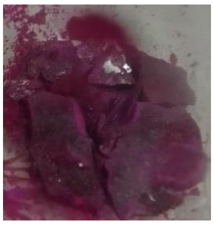

Following a literature known procedure [7] 3,6-diphenyl-1,2,4,5-tetrazine (**S1**, 1.40 g, 6.00 mmol), NIS (1.35 g, 6.00 mmol) and $Pd(OAc)_2$ (134 mg, 600 µmol) were dissolved in AcOH (48 mL) and stirred for 25 min under microwave irradiation (1200 W) at 100 °C. The reaction was cooled to room temperature, water (200 mL) was added and the mixture was extracted with EtOAc (3 × 200 mL). The combined organic layers were dried over Na_2SO_4, filtered and the solvent was removed under reduced pressure. The residue was purified via column chromatography on silica (Cy 98:2 EtOAc) to obtain 3-(2-iodophenyl)-6-phenyl-1,2,4,5-tetrazine (**S2a**, 808 mg, 2.24 mmol, 37%) as a purple solid and 3,6-bis(2-iodophenyl)-1,2,4,5-tetrazine (**S2b**, 378 mg, 778 µmol, 13%) as a purple solid (more polar).

S2a

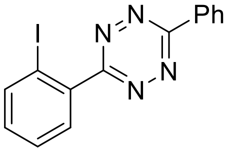

^{1}H NMR (600 MHz, $CDCl_3$) δ (ppm) 8.73 – 8.70 (m, 2H), 8.12 (dd, *J* = 8.0, 1.2 Hz, 1H), 8.00 (dd, *J* = 7.7, 1.7 Hz, 1H), 7.70 – 7.62 (m, 3H), 7.61 (td, *J* = 7.6, 1.2 Hz, 1H), 7.29 (td, *J* = 7.7, 1.7 Hz, 1H). **^{13}C NMR** (151 MHz, $CDCl_3$) δ (ppm) 167.4, 163.3, 141.2, 133.1, 132.4, 131.6, 129.5, 129.4, 128.8, 128.5, 128.1, 95.7. **MS** (ESI+) *m/z* 360.9 [M+H]$^+$. **IR** (ATR) ν (cm^{-1}) 3027, 2970, 1738, 1431, 1217, 909, 766, 690. **M$_P$**: 102-103 °C. The data is in accordance with the literature. [8]

S2b

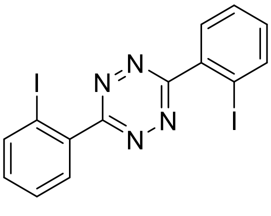

^{1}H NMR (600 MHz, $CDCl_3$) δ (ppm) 8.13 (dd, *J* = 8.0, 1.1 Hz, 2H), 8.08 (dd, *J* = 7.7, 1.6 Hz, 2H), 7.62 (td, *J* = 7.6, 1.1 Hz, 2H), 7.31 (ddd, *J* = 8.0, 7.4, 1.7 Hz, 2H). **^{13}C NMR** (151 MHz, $CDCl_3$) δ (ppm) 166.8, 141.2, 137.1, 132.8, 132.0, 128.8, 96.1. **MS** (ESI+) *m/z* 486.8 [M+H]$^+$. **IR** (ATR) ν (cm^{-1}) 3016, 1739, 1585, 1379, 1217, 1010, 892, 750, 713. **M$_P$**: 122-123 °C. The data is in accordance with the literature. [8]

5*H*-5λ^3-Benzo[4,5][1,2]iodazolo[2,3-*d*]tetrazol-5-ol (1a)

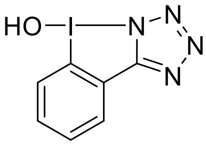

Following a known procedure [9] iodoarene **6** (1.63 g, 6.00 mmol) and Oxone monohydrate (1.85 g, 3.00 mmol) were suspended in water (12 mL) and the mixture was stirred at 75 °C for 4 h. The reaction was filtered at room temperature, washed with water (2 × 10 mL), MeCN (2 × 10 mL) and

Et_2O (10 mL) over an air steam (no high vacuum) to obtain **1a** (1.50 g, 5.21 mmol, 87%) as a colorless powder.

^{1}H NMR (600 MHz, DMSO-*d*$_6$) δ (ppm) 8.96 (s, 1H), 8.27 (d, *J* = 7.5 Hz, 1H), 7.98 (d, *J* = 8.3 Hz, 1H), 7.82 (t, *J* = 7.8 Hz, 1H), 7.77 (t, *J* = 7.4 Hz, 1H). **^{13}C NMR** (151 MHz, DMSO-*d*$_6$) δ (ppm) 160.1, 132.0, 131.2, 127.4, 127.2, 126.0, 121.3. **HRMS** (ESI+) Calculated for $C_7H_6IN_4O^+$ $[M+H]^+$ *m/z* 288.9581, found *m/z* 288.9579. **IR** (ATR) v (cm^{-1}) 2843, 2409, 1456, 1425, 1217, 1110, 1066, 996, 776, 736. **M$_P$**: 222-224 °C (decomposition). Caution. The compound in known to explode under rapid heating! The data is in accordance with the literature. [9]

(2-(2*H*-Tetrazol-5-yl)phenyl)(hydroxy)-*λ*^{3}-iodaneyl triflate (1b)

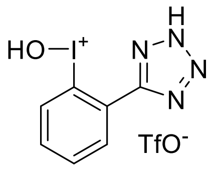

Following a modified literature procedure [5] iodoarene **6** (544 mg, 2.00 mmol) and *m*CPBA (85%, 449 mg, 2.20 mmol) were dissolved in DCM (10 mL) and TfOH (264 µL, 3.00 mmol) was added. The reaction was stirred at 40 °C for 2 h, stored at 4 °C for 30 min, filtered, washed with Et_2O (2 × 2 mL) and dried to obtain **1b** (623 mg, 1.42 mmol, 71%) as a colorless solid.

^{1}H NMR (600 MHz, DMSO-*d*$_6$) δ (ppm) 8.26 (dd, *J* = 7.5, 1.6 Hz, 1H), 7.97 (d, *J* = 8.3 Hz, 1H), 7.81 (td, *J* = 8.2, 7.8, 1.6 Hz, 1H), 7.76 (t, *J* = 7.4 Hz, 1H). **^{13}C NMR** (151 MHz, DMSO-*d*$_6$) δ (ppm) 160.1, 132.1, 131.2, 127.5, 127.2, 125.9, 121.3, 120.7 (q, *J* = 322.4 Hz). **^{19}F NMR** (565 MHz, DMSO-*d*$_6$) δ (ppm) -77.7. **HRMS** (ESI+) Calculated for $C_7H_6IN_4O^+$ $[M-OTf]^+$ *m/z* 288.95809, found *m/z* 288.95792. **IR** (ATR) v (cm^{-1}) 3084, 2915, 2765, 1604, 1473, 1269, 1212, 1020, 984, 739. **M$_P$**: 155 °C (slow explosion!).

Hydroxy(2-(6-phenyl-1,2,4,5-tetrazin-3-yl)phenyl)-*λ*^{3}-iodaneyl triflate (1c)

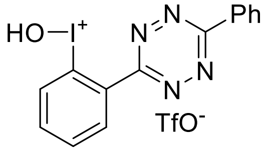

Following a modified literature procedure [5] iodoarene **S2a** (360 mg, 1.00 mmol) and *m*CPBA (85%, 225 mg, 1.10 mmol) were dissolved in DCM (7 mL) and TfOH (88.2 µL, 1.00 mmol) was added. The reaction was stirred at room temperature for 2 h, stored at 4 °C for 30 min, filtered, washed with Et_2O (2 × 2 mL) and dried to obtain **1c** (476 mg, 905 µmol, 91%) as red solid.

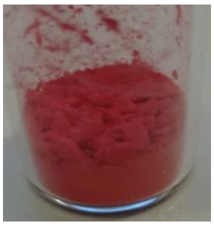

^{1}H NMR (600 MHz, CD_3CN) δ (ppm) 9.13 (dd, *J* = 7.8, 1.4 Hz, 1H), 8.63 (dd, *J* = 8.4, 1.3 Hz, 2H), 8.25 – 8.16 (m, 2H), 8.04 (ddd, *J* = 8.0, 6.5, 1.8 Hz, 1H), 7.83 (t, *J* = 7.4 Hz, 1H), 7.76 (t, *J* = 7.7 Hz, 2H). **^{13}C NMR** (151 MHz, CD_3CN) δ (ppm) 166.7, 159.3, 138.6, 135.6, 133.3, 131.8, 131.1, 131.0, 129.7, 128.5, 117.9 (one signal could not be detected and is probably overlapping with the solvent signal). **^{19}F NMR** (565 MHz, CD_3CN) δ (ppm) -77.7. **HRMS** (ESI+) Calculated for $C_{14}H_{10}IN_4O^+$ $[M-OTf]^+$

m/z 376.9894, found *m/z* 376.9883. **IR** (ATR) ν (cm^{-1}) 3016, 1737, 1445, 1390, 1217, 1154, 1025, 742. **M$_P$**: 165-166 °C (decomposition).

Hydroxy(2-(6-phenyl-1,2,4,5-tetrazin-3-yl)phenyl)-λ³-iodaneyl tosylate (1d)

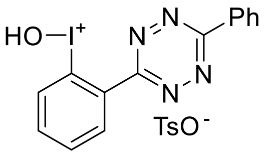

Following a modified literature procedure [5] iodoarene **S2a** (54.0 mg, 150 µmol) and *m*CPBA (85%, 33.7 mg, 165 µmol) were dissolved in DCM (1 mL) and TfOH (13.3 µL, 150 µmol) was added. The reaction was stirred at room temperature for 2 h, stored at 4 °C for 30 min, filtered, washed with Et_2O (2 x 2 mL) and dried to obtain **1d** (66.1 mg, 121 µmol, 80%) as a red solid.

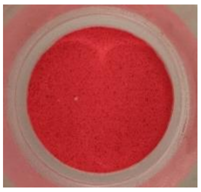

^{1}H NMR (600 MHz, CD_3CN) δ (ppm) 9.11 (d, *J* = 7.7 Hz, 1H), 8.62 (d, *J* = 7.3 Hz, 2H), 8.25 – 8.13 (m, 2H), 8.02 (ddd, *J* = 8.0, 6.1, 2.0 Hz, 1H), 7.82 (t, *J* = 7.4 Hz, 1H), 7.75 (t, *J* = 7.7 Hz, 2H), 7.68 (d, *J* = 8.0 Hz, 2H), 7.24 (d, *J* = 7.9 Hz, 2H), 2.36 (s, 3H). **^{13}C NMR** (151 MHz, CD_3CN) δ (ppm) 166.9, 159.4, 142.7, 141.6, 138.7, 135.7, 133.4, 131.9, 131.2, 131.1, 130.1, 129.8, 128.6, 126.9, 118.0, 21.4 (one signal could not be detected and is probably overlapping with the solvent signal). **HRMS** (ESI+) Calculated for $C_{14}H_{10}IN_4O^+$ [M-OTf]$^+$ *m/z* 376.9894, found *m/z* 376.9893. **IR** (ATR) ν (cm^{-1}) 3016, 1739, 1448, 1366, 1217, 1034, 1008, 685. **M$_P$**: 139-140 °C (decomposition).

((1,2,4,5-Tetrazine-3,6-diyl)bis(2,1-phenylene))bis(hydroxy-λ³-iodanediyl) bistriflate (1e)

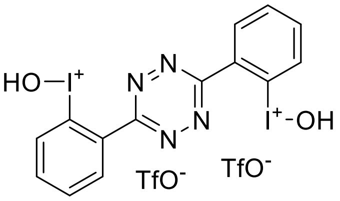

Following a modified literature procedure [5] iodoarene **S2b** (97.2 mg, 200 µmol) and *m*CPBA (85%, 89.8 mg, 440 µmol) were dissolved in MeCN (1 mL) and TfOH (88.4 µL, 440 µmol) was added. The reaction was stirred at room temperature for 2 h. Et_2O (2 mL) was added and the suspension was filtered, washed with Et_2O (2 × 2 mL) and dried to obtain **1e** (161 mg, 197 µmol, 99%) as a yellow solid.

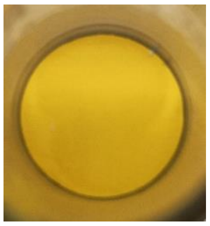

^{1}H NMR (600 MHz, CD_3CN) δ (ppm) 9.03 (dd, *J* = 7.7, 1.6 Hz, 1H), 8.29 (ddd, *J* = 8.7, 7.3, 1.6 Hz, 1H), 8.21 (d, *J* = 8.3 Hz, 1H), 8.12 – 8.02 (m, 2H). **^{13}C NMR** (151 MHz, CD_3CN) δ (ppm) 162.2, 140.0, 133.8, 132.8, 128.6, 127.5, 119.0. **^{19}F NMR** (565 MHz, CD_3CN) δ (ppm) -79.3. Measurement of ^{13}C NMR was challenging due to poor solubility and slow decomposition in CD_3CN and fast decomposition in DMSO-*d*$_6$ and CD_3OD. The following data were recorded with a few additional drops of trifluoroacetic acid. **^{1}H NMR** (600 MHz, CD_3CN) δ (ppm) 9.02 (d, *J* = 7.7 Hz, 1H), 8.28

(t, J = 7.9 Hz, 1H), 8.21 (d, J = 8.4 Hz, 1H), 8.08 (t, J = 7.5 Hz, 1H). **^{13}C NMR** (151 MHz, CD_3CN) δ (ppm) 162.4, 140.2, 134.0, 133.0, 128.8, 127.6, 119.0. **^{19}F NMR** (565 MHz, CD_3CN) δ (ppm) -79.6. **HRMS** (ESI+) Calculated for $C_{14}H_9I_2N_2O_2^+$ [M-2OTf-H-N_2]$^+$ *m/z* 490.8748, found *m/z* 490.8739. (One of the iodine atoms is reduced to I(I) and in the tetrazine moiety N_2 is substituted by an oxygen.) **IR** (ATR) ν (cm^{-1}) 3067, 1589, 1489, 1406, 1280, 1240, 1155, 1024, 765, 709. **M$_p$**: 160-161 °C.

((1,2,4,5-Tetrazine-3,6-diyl)bis(2,1-phenylene))bis(mesityliodonium) bistriflate (S3)

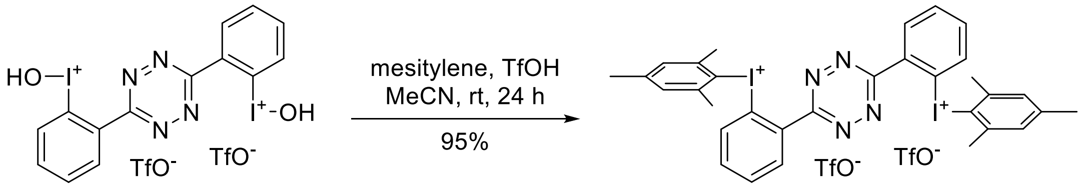

1e **S3**

Note: No HRMS could be measured of compound **1e**. To proof the successful synthesis of **1e** this iodane was transferred into the bismesitylene salt **S3**.

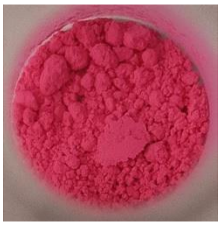

To a mixture of **1i** (81.2 mg, 100 µmol) and mesitylene (43.2 µL, 240 µmol) in MeCN (0.5 mL) was added TfOH (26.5 µL, 300 µmol) and the reaction was stirred for 24 h at room temperature. The suspension was filtered, washed with MeCN (1 mL), Et_2O (1 mL) and was dried to obtain **S3** (97.2 mg, 950 µmol, 95%) as pink solid.

^{1}H NMR (601 MHz, DMSO-d_6) δ (ppm) 9.00 (d, J = 7.7 Hz, 1H), 8.01 (t, J = 7.6 Hz, 1H), 7.85 (t, J = 7.9 Hz, 1H), 7.44 (s, 2H), 7.23 (d, J = 8.3 Hz, 1H), 2.58 (s, 6H), 2.44 (s, 3H). **^{13}C NMR** (151 MHz, DMSO-d_6) δ (ppm) 161.38, 144.72, 142.87, 137.16, 132.50, 132.25, 130.94, 130.37, 130.27, 121.08, 120.7 (q, J = 322.3 Hz), 111.79, 26.18, 20.82. **^{19}F NMR** (565 MHz, DMSO-d_6) δ (ppm) -77.8. **HRMS** (ESI+) Calculated for $C_{32}H_{30}I_2N_4^{2+}$ [M-2OTf]$^{2+}$ *m/z* 362.0275, found *m/z* 362.0271. **IR** (ATR) ν (cm^{-1}) 3086, 2958, 2919, 1588, 1444, 1392, 1256, 1142, 1029, 769, 704. **M$_p$**: 256 - 257 °C (slow explosion!).

3 Optimization data

Temperature

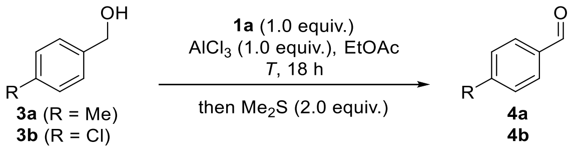

Substrate	R	*T* [°C]	Yield **4a**/**4b** [%]
**3a**	Me	rt	21
**3a**	Me	50	55
**3a**	**Me**	**60**	**61**
**3a**	Me	70	58
**3a**	**Me**	**60**	**65**[a]
**3b**	Cl	rt	8
**3b**	Cl	50	39
**3b**	**Cl**	**60**	**43**
**3b**	Cl	70	26
**3b**	**Cl**	**60**	**39**[a]

Reaction conditions: Iodane **1a** (100 µmol, 28.8 mg), alcohol **3a** (100 µmol, 12.2 mg) or **3b** (100 µmol, 14.3 mg), respectively and $AlCl_3$ (100 µmol, 13.3 mg) were stirred with EtOAc (1 mL) as solvent for 18 h at the given temperature. Me_2S (200 µmol, 14.7 µL) was added, stirred for 5 min and then the solvent was removed under reduced pressure. The residue was dissolved in DMSO-d_6 and the yield was determined using tetraethylsilane as standard. a: 2.5 h reaction time.

Additive and solvent

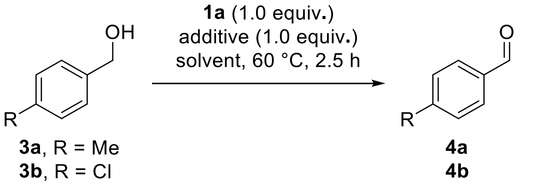

Entry	Additive	Solvent	Yield [%]	
			**4a**	**4b**
1	$AlCl_3$	EtOAc	65	39
2	TsOH·H_2O	EtOAc	1	1
3	NaOTs	EtOAc	1	1
4	TBAF	EtOAc	9	19
5	TBACl	EtOAc	67	62
6	TBABr	EtOAc	58	47
7	TBAI	EtOAc	40	36
8	NH_4Cl	EtOAc	37	26
9	HCl	EtOAc	82	44
10	HCl	TFE	1	-
11	HCl	MeCN	72	-
12	HCl	Toluene	20	-
13	HCl	$CDCl_3$	49	-
14	HCl	MeOH	48	-
15	HCl	EtOAc/MeCN (1:1)	79	-
16	HCl	H_2O	1	-
17	TBACl	MeCN	64	69
18	TBACl	$CDCl_3$	-	32
19	TBACl	EtOAc/MeCN (1:1)	-	55
20	TBACl	H_2O	-	2

Reaction conditions: **1a** (100 µmol), **3a**/**3b** (100 µmol) and the additive (100 µmol) were stirred in the given solvent (1 mL) at 60 °C for 2.5 h and quenched with Me_2S (200 µmol). The yield was determined via ^1H NMR using tetraethylsilane as an internal standard.

Concentration

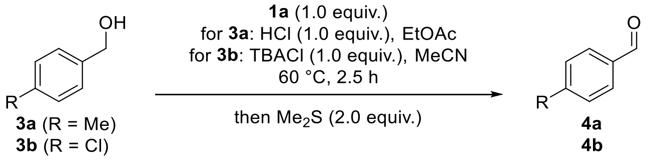

Substrate	R	*c* [mM]	**4a** [%]	**3a** [%]
**3a**	Me	1.00	81	2
**3a**	Me	0.50	78	3
**3a**	Me	**0.20**	**80/78**	**2/2**
**3a**	Me	0.10	76	2
**3a**	Me	0.05	68	15
**3a**	Me	0.02	47	45
**3b**	Cl	0.05	65	30
**3b**	Cl	0.10	72/68	23/19
**3b**	Cl	**0.20**	**81**	**18**
**3b**	Cl	1.00	75	18

Reaction conditions: Iodane **1a** (100 µmol, 28.8 mg), alcohol **3a** (100 µmol, 12.2 mg) or **3b** (100 µmol, 14.3 mg), respectively and additive (100 µmol) were stirred with EtOAc or MeCN as solvent for 2.5 h at 60 °C. Me_2S (200 µmol, 14.7 µL) was added, stirred for 5 min and then the solvent was removed under reduced pressure. The residue was dissolved in DMSO-d_6 and the yield was determined using tetraethylsilane as standard.

Equivalents of additive TBACl

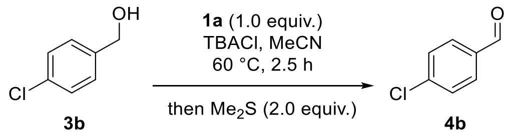

equiv nBu$_4$NCl	**4b** [%]	**3b** [%]
0.5	47	45
**1.0**	**72/68**	**23/19**
1.25	73	25
1.5	69	30
2.0	73	27

Iodane **1a** (100 µmol, 28.8 mg), alcohol **3b** (100 µmol, 14.3 mg), and TBACl were stirred with MeCN (0.5 mL) as solvent for 2.5 h at 60 °C. Me_2S (200 µmol, 14.7 µL) was added, stirred for 5 min and then the solvent was removed under reduced pressure. The residue was dissolved in DMSO-d_6 and the yield was determined using tetraethylsilane as standard.

Equivalents of iodane

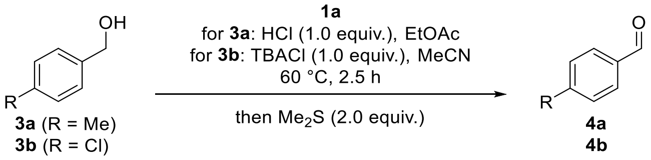

Substrate	R	equiv of **1a**	Yield **4a**/**4b** [%]
**3a**	Me	1.0	84
**3a**	Me	1.2	86
**3a**	**Me**	**1.4**	**90**
**3a**	Me	1.6	86
**3b**	Cl	1.0	70
**3b**	Cl	1.2	71
**3b**	**Cl**	**1.4**	**78**
**3b**	Cl	1.6	69

Reaction conditions: Iodane **1a,** alcohol **3a** (100 µmol, 12.2 mg) or **3b** (100 µmol, 14.3 mg), respectively and additive (100 µmol) were stirred with EtOAc or MeCN (0.5 mL) as solvent for 2.5 h at 60 °C. Me_2S (200 µmol, 14.7 µL) was added, stirred for 5 min and then the solvent was removed under reduced pressure. The residue was dissolved in DMSO-d_6 and the yield was determined using tetraethylsilane as standard.

4 Oxidation of benzylic alcohols

4.1 General procedure for oxidation of electron-rich alcohols (GP A)

Iodane **1a** (700 µmol, 201 mg, 1.40 equiv) and alcohol (500 µmol, 1.00 equiv) were dissolved/suspended in EtOAc (2.5 mL) in a screw cap vial and aq. HCl (37%, 500 µmol, 41.6 µL, 1.00 equiv) was added. The reaction was stirred for 2.5 h at 60 °C, then Me_2S (1.40, 104 µL, 2.80 equiv) was added and stirring was continued for 5 min. Then, the solvent was removed under reduced pressure and the reaction was purified on column chromatography with silica to obtain the pure aldehyde.

4.2 General procedure for oxidation of electron-poor alcohols (GP B)

Iodane **1a** (700 µmol, 201 mg, 1.40 equiv) and alcohol (500 µmol, 1.00 equiv) were dissolved/suspended in MeCN (2.5 mL) in a screw cap vial and TBACl (500 µmol, 139 mg, 1.00 equiv) was added. The reaction was stirred for 2.5 h at 60 °C, then Me_2S (1.40, 104 µL, 2.80 equiv) was added and stirring was continued for 5 min. Then, the solvent was removed under reduced pressure and the reaction was purified on column chromatography with silica to obtain the pure aldehyde.

4.3 Substrate scope

4-Methylbenzaldehyde (4a)

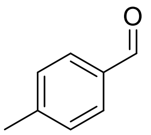

Following **GP A** the aldehyde **4a** (50.2 mg, 418 µmol, 84%) was obtained as a colorless solid. Additionally, the iodoarene **6** (172 mg, 633 µmol, 90%) was reisolated as a colorless solid.

^{1}H NMR (600 MHz, $CDCl_3$) δ (ppm) 9.97 (s, 1H), 7.78 (d, *J* = 8.1 Hz, 2H), 7.33 (d, *J* = 7.8 Hz, 2H), 2.44 (s, 3H). **^{13}C NMR** (151 MHz, $CDCl_3$) δ (ppm) 192.1, 145.7, 134.4, 130.0, 129.9, 22.0. **MS** (EI) *m/z* 119.09 $[M-H]^+$. **IR** (ATR) ν (cm^{-1}) 3027, 2970, 2822, 2732, 1701, 1603, 1365, 1207, 1167, 846, 806, 757. **M$_P$**: 109-110 °C. The data is in accordance with the literature. [10]

4-Chlorobenzaldehyde (4b)

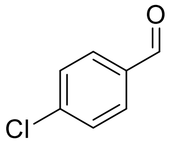

Following **GP B** the aldehyde **4b** (57.2 mg, 407 µmol, 81%) was obtained as a colorless solid.

^{1}H NMR (600 MHz, $CDCl_3$) δ (ppm) 9.98 (s, 1H), 7.82 (d, *J* = 8.4 Hz, 2H), 7.51 (d, *J* = 8.4 Hz, 2H). **^{13}C NMR** (151 MHz, $CDCl_3$) δ (ppm) 191.0, 141.1, 134.9, 131.0, 129.6. **MS** (EI) *m/z* 138.95 $[M-H]^+$. **IR** (ATR) ν (cm^{-1}) 2810, 2551, 1674, 1590, 1421, 1281, 1091, 923, 851, 758. **M_P**: 46-47 °C. The data is in accordance with the literature. [11]

4-Bromobenzaldehyde (4c)

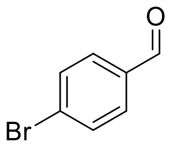

Following **GP B** the aldehyde **4c** (81.1 mg, 438 µmol, 88%) was obtained as a colorless solid.

^{1}H NMR (600 MHz, $CDCl_3$) δ (ppm) 9.97 (s, 1H), 7.74 (d, *J* = 8.5 Hz, 2H), 7.67 (d, *J* = 8.5 Hz, 2H). **^{13}C NMR** (151 MHz, $CDCl_3$) δ (ppm) 191.2, 135.2, 132.6, 131.1, 129.9. **MS** (EI) *m/z* 182.90 $[M-H]^+$. **IR** (ATR) ν (cm^{-1}) 2956, 1678, 1585, 1279, 1065, 1010, 811, 755. **M_P**: 51-52 °C. The data is in accordance with the literature. [11]

4-Iodobenzaldehyde (4d)

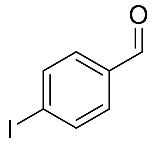

Following **GP A** the aldehyde **4d** (87.2 mg, 376 µmol, 75%) was obtained as a colorless solid.

^{1}H NMR (600 MHz, $CDCl_3$) (ppm) δ 9.95 (s, 1H), 7.90 (d, *J* = 8.3 Hz, 2H), 7.58 (d, *J* = 8.3 Hz, 2H). **^{13}C NMR** (151 MHz, $CDCl_3$) δ (ppm) 191.5, 138.5, 135.7, 130.9, 103.0. **MS** (EI) *m/z* 231.95 $[M]^{+\bullet}$. **IR** (ATR) ν (cm^{-1}) 2966, 1657, 1255, 1060, 1015, 821. **M_P**: 83-84 °C. The data is in accordance with the literature. [11]

4-Nitrobenzaldehyde (4e)

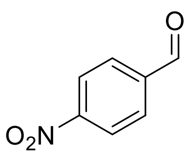

Following **GP B** the aldehyde **4e** (51.8 mg, 343 µmol, 69%) was obtained as a colorless solid.

^{1}H NMR (600 MHz, $CDCl_3$) δ (ppm) 10.15 (s, 1H), 8.39 (d, *J* = 8.7 Hz, 2H), 8.07 (d, *J* = 8.7 Hz, 2H). **^{13}C NMR** (151 MHz, $CDCl_3$) δ (ppm) 190.4, 151.2, 140.2, 130.6, 124.4. **MS** (EI) *m/z* 150.96 $[M]^{+\bullet}$. **IR** (ATR) ν (cm^{-1}) 3211, 2955, 1703, 1525, 1342, 1194, 848, 812, 737, 677. **M_P**: 105-106 °C. The data is in accordance with the literature. [11]

4-Formylbenzonitrile (4f)

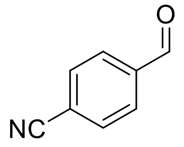

Following **GP B** the aldehyde **4f** (52.2 mg, 398 μmol, 80%) was obtained as a colorless solid.

^{1}**H NMR** (600 MHz, $CDCl_3$) δ (ppm) 10.10 (s, 1H), 8.00 (d, *J* = 8.3 Hz, 2H), 7.85 (d, *J* = 8.2 Hz, 2H). ^{13}**C NMR** (151 MHz, $CDCl_3$) δ (ppm) 190.7, 138.9, 133.1, 130.0, 117.8, 117.8. **MS** (EI) *m/z* 130.02 $[M-H]^+$. **IR** (ATR) ν (cm^{-1}) 3ß94, 2850, 2745, 2229, 1700, 1607, 1571, 1385, 1296, 1201, 1172, 828, 737. **M$_P$**: 100-101 °C. The data is in accordance with the literature. [11]

2-Iodobenzaldehyde (4g)

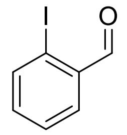

Following **GP B** the aldehyde **4g** (49.5 mg, 213 μmol, 43%) was obtained as a colorless solid.

^{1}**H NMR** (600 MHz, $CDCl_3$) δ (ppm) 10.06 (s, 1H), 7.95 (dd, *J* = 7.9, 1.1 Hz, 1H), 7.87 (dd, *J* = 7.7, 1.8 Hz, 1H), 7.46 (t, *J* = 7.5 Hz, 1H), 7.28 (td, *J* = 7.6, 1.8 Hz, 1H). ^{13}**C NMR** (151 MHz, $CDCl_3$) δ (ppm) 195.9, 140.8, 135.6, 135.3, 130.4, 128.8, 100.8. **IR** (ATR) ν (cm^{-1}) 2849, 2746, 1682, 1578, 1388, 1261, 1200, 1016, 821, 747, 671. **MS** (EI) m/z = 231.96 $[M]^{+\bullet}$. **M$_P$**: 36 - 37 °C. The data is in accordance with the literature. [12]

[1,1'-Biphenyl]-2-carbaldehyde (4h)

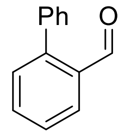

Following **GP B** the aldehyde **4h** (77.3 mg, 424 μmol, 85%) was obtained as a colorless solid.

^{1}**H NMR** (600 MHz, $CDCl_3$) δ (ppm) 9.99 (d, *J* = 0.9 Hz, 1H), 8.04 (dd, *J* = 7.8, 1.4 Hz, 1H), 7.65 (td, *J* = 7.5, 1.4 Hz, 1H), 7.56 – 7.42 (m, 5H), 7.39 (dd, *J* = 8.0, 1.6 Hz, 2H). ^{13}**C NMR** (151 MHz, $CDCl_3$) δ (ppm) 192.6, 146.1, 137.8, 133.8, 133.7, 130.9, 130.2, 128.5, 128.2, 127.9, 127.7. **MS** (EI) *m/z* 180.94 $[M-H]^+$. **IR** (ATR) ν (cm^{-1}) 3059, 2843, 2750, 1687, 1595, 1194, 745, 700. **M$_P$**: 47-48 °C. The data is in accordance with the literature. [13]

2-(Trifluoromethyl)benzaldehyde (4j)

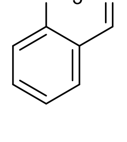

Following **GP B** the aldehyde **4j** (48.9 mg, 264 μmol, 53%) was obtained as a colorless oil.

^{1}**H NMR** (600 MHz, $CDCl_3$) δ (ppm) 10.41 (q, *J* = 2.1 Hz, 1H), 8.30 – 7.98 (m, 1H), 7.89 – 7.75 (m, 1H), 7.75 – 7.57 (m, 2H). ^{13}**C NMR** (151 MHz, $CDCl_3$) δ (ppm) 189.1, 133.9, 133.8, 132.5, 131.2 (q, *J* = 32.2 Hz), 129.2, 126.3 (q, *J* = 5.7 Hz), 123.9 (q, *J* = 274.4 Hz). ^{19}**F NMR** (565 MHz, $CDCl_3$) δ (ppm) -55.55 (d, *J* = 2.2 Hz). **MS** (EI) *m/z* 172.99 $[M-H]^+$. **IR**

(ATR) ν (cm^{-1}) 2895, 2657, 1702, 1416, 1275, 1126, 1037, 892, 764, 677. The data is in accordance with the literature. [14]

3-(Trifluoromethyl)benzaldehyde (4k)

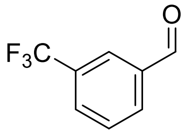

Following **GP B** the aldehyde **4k** (73.2 mg, 420 µmol, 84%) was obtained as a colorless oil.

^{1}H NMR (600 MHz, $CDCl_3$) δ (ppm) 10.08 (s, 1H), 8.15 (d, *J* = 2.1 Hz, 1H), 8.08 (d, *J* = 7.7 Hz, 1H), 7.89 (d, *J* = 7.3 Hz, 1H), 7.70 (t, *J* = 7.7 Hz, 1H). **^{13}C NMR** (151 MHz, $CDCl_3$) δ (ppm) 190.9, 136.9, 132.8, 132.0 (q, *J* = 33.3 Hz), 131.0 (q, *J* = 3.6 Hz), 129.9, 126.6 (q, *J* = 3.8 Hz), 123.7 (q, *J* = 272.6 Hz). **^{19}F NMR** (565 MHz, $CDCl_3$) δ (ppm) -63.0. **MS** (EI) *m/z* 173.00 $[M-H]^+$. **IR** (ATR) ν (cm^{-1}) 2928, 2850, 2739, 1704, 1326, 1166, 1122, 1069, 802, 694. The data is in accordance with the literature. [15]

4-(Trifluoromethyl)benzaldehyde (4l)

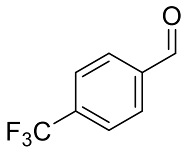

Following **GP B** the aldehyde **4l** (61.8 mg, 355 µmol, 71%) was obtained as a colorless oil.

^{1}H NMR (600 MHz, $CDCl_3$) δ (ppm) 10.11 (s, 1H), 8.01 (d, *J* = 7.9 Hz, 2H), 7.81 (d, *J* = 8.0 Hz, 2H). **^{13}C NMR** (151 MHz, $CDCl_3$) δ (ppm) 191.3, 138.8, 135.8 (q, *J* = 32.7 Hz), 130.1, 126.3 (q, *J* = 3.7 Hz), 123.6 (q, *J* = 272.9 Hz). **MS** (EI) *m/z* 172.98 $[M-H]^+$. **IR** (ATR) ν (cm^{-1}) 2836, 2741, 1707, 1513, 1424, 1389, 1320, 1166, 1123, 1062, 1016, 832, 759. The data is in accordance with the literature. [14]

2,6-Dichlorobenzaldehyde (4m)

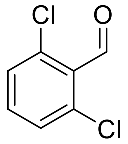

Following **GP B** the aldehyde **4m** (34.2 mg, 195 µmol, 39%) was obtained as a colorless solid.

^{1}H NMR (600 MHz, $CDCl_3$) δ (ppm) 10.49 (s, 1H), 8.06 – 6.89 (m, 3H). **^{13}C NMR** (151 MHz, $CDCl_3$) δ (ppm) 188.9, 137.0, 133.7, 130.6, 129.9. **MS** (EI) *m/z* 172.92 $[M-H]^+$. **IR** (ATR) ν (cm^{-1}) 3093, 2892, 1698, 1576, 1434, 1185, 1093, 775, 660. **M$_P$**: 68 - 69 °C. The data is in accordance with the literature. [16]

2-Naphthaldehyde (4n)

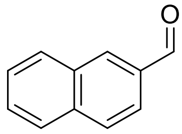

Following **GP B** the aldehyde **4n** (34.7 mg, 222 μmol, 44%) was obtained as a colorless solid.

^{1}H NMR (600 MHz, $CDCl_3$) δ (ppm) 10.16 (s, 1H), 8.34 (d, *J* = 1.5 Hz, 1H), 8.01 (d, *J* = 8.2 Hz, 1H), 7.98 – 7.87 (m, 3H), 7.65 (ddd, *J* = 8.2, 6.8, 1.3 Hz, 1H), 7.59 (ddd, *J* = 8.1, 6.8, 1.3 Hz, 1H). **^{13}C NMR** (151 MHz, $CDCl_3$) δ (ppm) 192.4, 136.6, 134.7, 134.3, 132.8, 129.7, 129.3, 129.2, 128.2, 127.2, 122.9. **MS** (EI) *m/z* 156.03 $[M]^{+\bullet}$ **IR** (ATR) ν (cm^{-1}) 3061, 2923, 2869, 1683, 1594, 1318, 1219, 989, 810, 752. **M_P**: 61 - 62 °C. The data is in accordance with the literature. [17]

Picolinaldehyde (4o)

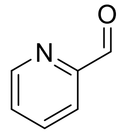

Following **GP B** the aldehyde **4o** (46.4 mg, 433 μmol, 87%) was obtained as a yellowish oil.

^{1}H NMR (600 MHz, $CDCl_3$) δ (ppm) 10.09 (d, *J* = 0.8 Hz, 1H), 8.80 (ddd, *J* = 4.8, 1.7, 0.9 Hz, 1H), 7.97 (dt, *J* = 7.7, 1.1 Hz, 1H), 7.89 (tdd, *J* = 7.7, 1.7, 0.9 Hz, 1H), 7.53 (ddd, *J* = 7.6, 4.7, 1.3 Hz, 1H). **^{13}C NMR** (151 MHz, $CDCl_3$) δ (ppm) 193.3, 152.7, 150.1, 137.0, 127.8, 121.6. **MS** (EI) *m/z* 107.00 $[M]^{+\bullet}$. **IR** (ATR) ν (cm^{-1}) 3015, 2970, 2840, 1710, 1365, 1216, 994, 737. The data is in accordance with the literature. [18]

Nicotinaldehyde (4p)

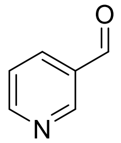

Following **GP B** the aldehyde **4p** (34.4 mg, 321 μmol, 64%) was obtained as a colorless oil.

^{1}H NMR (600 MHz, $CDCl_3$) δ (ppm) 10.12 (s, 1H), 9.08 (dd, *J* = 2.1, 0.9 Hz, 1H), 8.85 (dd, *J* = 4.9, 1.8 Hz, 1H), 8.18 (dt, *J* = 7.9, 2.0 Hz, 1H), 7.49 (ddt, *J* = 7.8, 4.8, 0.7 Hz, 1H). **^{13}C NMR** (151 MHz, $CDCl_3$) δ (ppm) 190.8, 154.9, 152.2, 136.0, 131.6, 124.3. **MS** (EI) *m/z* 107.01 $[M]^{+\bullet}$. **IR** (ATR) ν (cm^{-1}) 3381, 2840, 1698, 1588, 1427, 1210, 1024, 831, 700. The data is in accordance with the literature. [10]

***trans*-2-Phenylcyclopropane-1-carbaldehyde (4r)**

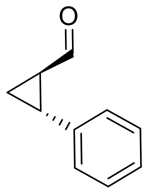

Following **GP B** the aldehyde **4r** (36.8 mg, 264 μmol, 53%) was obtained as a colorless oil.

^{1}H NMR (600 MHz, $CDCl_3$) δ (ppm) 9.33 (d, *J* = 4.6 Hz, 1H), 7.30 (t, *J* = 7.5 Hz, 2H), 7.23 (t, *J* = 7.4 Hz, 1H), 7.12 (d, *J* = 7.3 Hz, 2H), 2.63 (ddd, *J* = 9.2, 6.7, 4.0 Hz, 1H), 2.18 (dddd, *J* = 8.4, 5.2, 4.6, 4.0 Hz, 1H), 1.74 (dtd, *J* = 9.2, 5.1, 0.7 Hz, 1H), 1.53 (ddd,

J = 8.2, 6.7, 5.0 Hz, 1H). **^{13}C NMR** (151 MHz, $CDCl_3$) δ (ppm) 199.8, 139.1, 128.7, 126.9, 126.4, 33.9, 26.7, 16.5. **MS** (EI) *m/z* 145.03 $[M\text{-}H]^+$. **IR** (ATR) ν (cm^{-1}) 3030, 2832, 2730, 1695, 1604, 1498, 1443, 1180, 1079, 1055, 1028, 922, 755, 695. The data is in accordance with the literature. [19]

1-(*p*-Tolyl)ethan-1-one (4t)

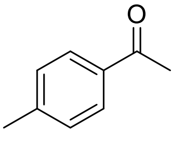

Following **GP A** the ketone **4t** (65.2 mg, 486 μmol, 97%) was obtained as a colorless oil.

^{1}H NMR (600 MHz, $CDCl_3$) δ (ppm) 7.85 (d, *J* = 8.3 Hz, 2H), 7.25 (dt, *J* = 8.0, 0.8 Hz, 2H), 2.57 (s, 3H), 2.40 (s, 3H). **^{13}C NMR** (151 MHz, $CDCl_3$) δ (ppm) 198.0, 144.0, 134.8, 129.3, 128.5, 26.6, 21.7. **MS** (EI) *m/z* 134.11 $[M]^{+\bullet}$. **IR** (ATR) ν (cm^{-1}) 3003, 2970, 2923, 1677, 1605, 1356, 1266, 953, 812. The data is in accordance with the literature. [17]

2,3-Dihydro-1*H*-inden-1-one (4u)

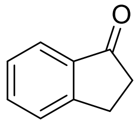

Following **GP A** the ketone **4u** (30.5 mg, 231 μmol, 46%) was obtained as a colorless solid.

^{1}H NMR (600 MHz, $CDCl_3$) δ (ppm) 7.76 (dt, *J* = 7.7, 1.0 Hz, 1H), 7.58 (td, *J* = 7.4, 1.2 Hz, 1H), 7.48 (dt, *J* = 7.7, 0.9 Hz, 1H), 7.37 (td, *J* = 7.4, 0.9 Hz, 1H), 3.23 – 2.96 (m, 2H), 2.71 – 2.67 (m, 2H). **^{13}C NMR** (151 MHz, $CDCl_3$) δ (ppm) 207.3, 155.3, 137.2, 134.7, 127.4, 126.8, 123.9, 36.4, 25.9. **MS** (EI) *m/z* 132.04 $[M]^{+\bullet}$. **IR** (ATR) ν (cm^{-1}) 3031, 2924, 2858, 1704, 1525, 1342, 812, 754. **M_P**: 40-41 °C. The data is in accordance with the literature. [16]

Carvone (4v)

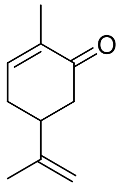

Following **GP B** the ketone **4v** (55.6 mg, 370 μmol, 74%) was obtained as a colorless oil.

^{1}H NMR (600 MHz, $CDCl_3$) δ (ppm) 6.75 (ddd, *J* = 5.9, 2.7, 1.4 Hz, 1H), 4.81 (t, *J* = 1.6 Hz, 1H), 4.76 (d, *J* = 1.6 Hz, 1H), 2.69 (ddt, *J* = 14.4, 10.6, 4.2 Hz, 1H), 2.59 (ddd, *J* = 16.0, 3.7, 1.6 Hz, 1H), 2.44 (dddt, *J* = 18.2, 5.9, 4.5, 1.4 Hz, 1H), 2.35 (dd, *J* = 16.0, 13.3 Hz, 1H), 2.28 (ddt, *J* = 18.3, 10.8, 2.5 Hz, 1H), 1.79 (dt, *J* = 2.6, 1.4 Hz, 3H), 1.75 (t, *J* = 1.1 Hz, 3H). **^{13}C NMR** (151 MHz, $CDCl_3$) δ (ppm) 199.9, 146.9, 144.7, 135.6, 110.6, 43.3, 42.6, 31.4, 20.7, 15.9. **MS** (EI) *m/z* 150.06 $[M]^{+\bullet}$. **IR** (ATR) ν (cm^{-1}) 2970, 2923, 2854, 1738, 1670, 1365, 1217, 1109, 891. The data is in accordance with the literature. [20]

5 Mechanistic studies

Indication for the formation of an alkoxy-NHI

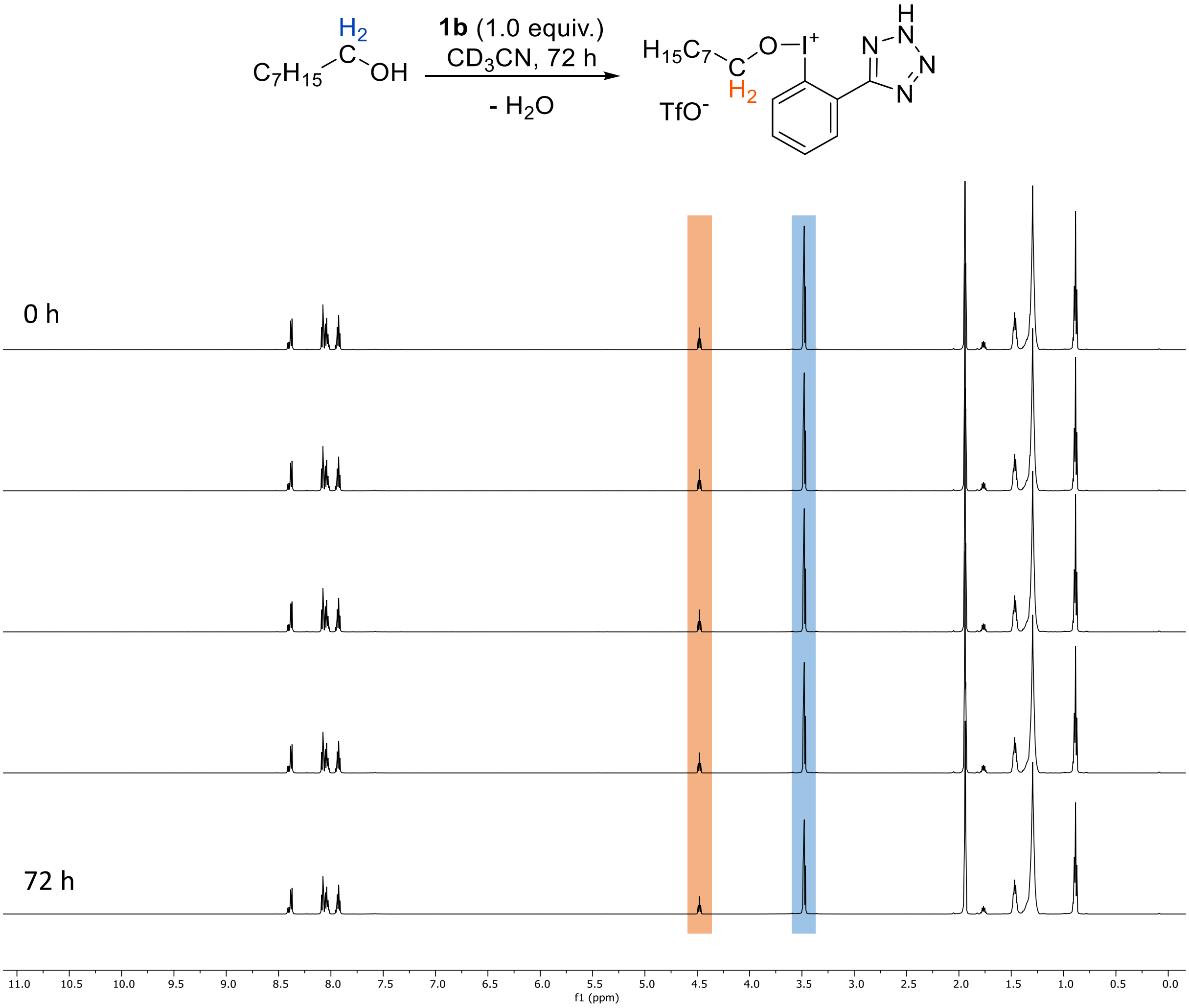

Figure S1: Reaction of the tetrazole-substituted (hydroxy)iodonium triflate **1b** with 1-octanol (**2**) leading to a significant downfield shift of the protons on the alpha-carbon from 3.48 ppm (blue) to 4.48 ppm (orange), indicating a bonding of the iodane to the alcohol and therefore resulting in a loss of electron density. Reaction conditions: An equimolar mixture of NHI **1b** (10.0 µmol) and alcohol **2** (10.0 µmol) were dissolved in CD_3CN (600 µL) and ^1H NMR spectra were recorded.

Indication of an activation of the iodane by chloride

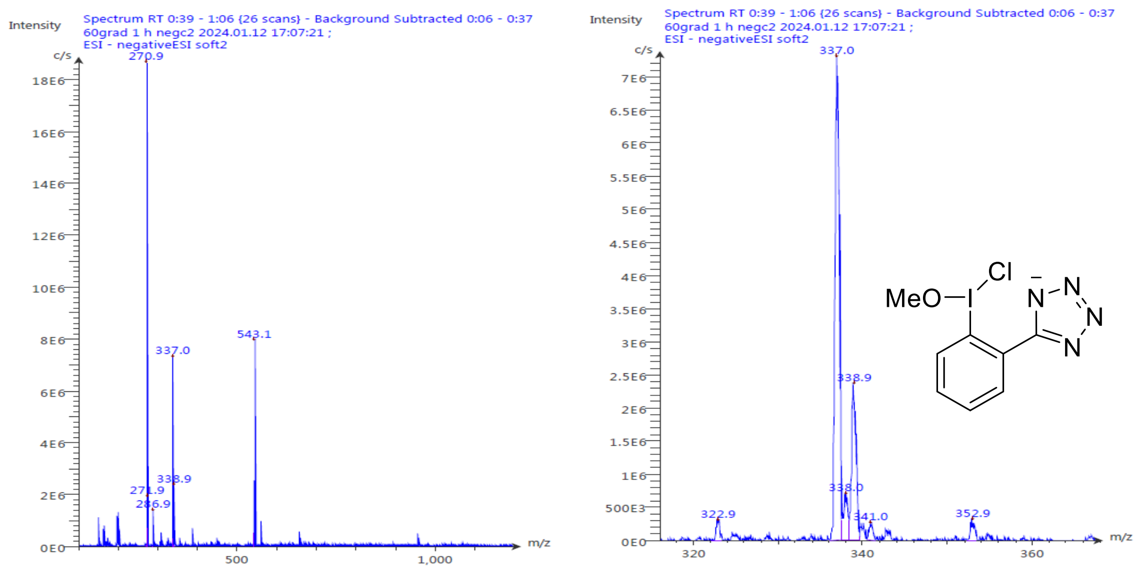

Figure S2: Indication of an activation of the iodane **1a** by formation of an I–Cl bonding. Reaction conditions: iodane **1a** (100 µmol, 28.8 mg) and aq. HCl (37%, 8.32 µL) were dissolved in EtOAc (500 µL), stirred for 1 h at 60 °C and an ESI(−) mass spectrum was recorded. Due to MeOH as solvent in the mass spectrometer a substitution of hydroxy with methoxy is possible. Note: Before heating the mixture also ESI(−) mass spectra were recorded, but no such species could be detected.

6 References

1. Armarego, W. L. F.; Chai, C. L. *Purification of Laboratory Chemicals-Six Edition;* Elsevier Inc., London, 2009.
2. G. M. Sheldrick. *J. Appl. Crystallogr.* **2009** (42), 339.
3. G. M. S. Sheldrick. *Acta Cryst.* **2015**, 3.
4. G. M. S. Sheldrick. *Acta Cryst.* **2008**, 112.
5. Vaish, A.; Sayala, K. D.; Tsarevsky, N. V. *Tetrahedron Lett.* **2019,** *60* (35), 150995.
6. Bachollet, Sylvestre P. J. T.; Vece, V.; McCracken, A. N.; Finicle, B. T.; Selwan, E.; Ben Romdhane, N.; Dahal, A.; Ramirez, C.; Edinger, A. L.; Hanessian, S. *ACS Med. Chem. Lett.* **2020,** *11* (5), 686–690.
7. Mboyi, C. D.; Testa, C.; Reeb, S.; Genc, S.; Cattey, H.; Fleurat-Lessard, P.; Roger, J.; Hierso, J.-C. *ACS Catal.* **2017,** *7* (12), 8493–8501.
8. Testa, C.; Gigot, É.; Genc, S.; Decréau, R.; Roger, J.; Hierso, J.-C. *Angew. Chem. Int. Ed.* **2016,** *55* (18), 5555–5559.
9. Kumar, R.; Sayala, K. D.; Camdzic, L.; Siegler, M.; Vaish, A.; Tsarevsky, N. *ChemRxiv* **2021**. DOI:10.26434/chemrxiv-2021-gsp7q-v2.
10. Zhang, G.; Wang, Y.; Wen, X.; Ding, C.; Li, Y. *Chem. Commun.* **2012,** *48* (24), 2979–
11. Du Chen; Xu, L.; Yu, Y.; Mo, Q.; Qi, X.; Liu, C. *Angew. Chem. Int. Ed.* **2023,** *62* (2),
12. Boelke, A.; Lork, E.; Nachtsheim, B. J. *Chem. Eur. J.* **2018,** *24* (70), 18653–18657.
13. Cheng, X.; Li, W.; Nie, R.; Ma, X.; Sang, R.; Guo, L.; Wu, Y. *Adv. Synth. Catal.* **2017,** *359* (3), 454–466.
14. Nadhagopal, S.; Dolas, Z.; Silamkoti, A.; Gupta, A.; Mathur, A.; Karmakar, S. *Synlett* **2023,** *34* (15), 1809–1813.
15. Fan, Q.; Liu, D.; Xie, Z.; Le, Z.; Zhu, H.; Song, X. *J. Org. Chem.* **2023,** *88* (20), 14559–14570.
16. Jiang, L.-Y.; Ming, J.-J.; Wang, L.-Y.; Jiang, Y.-Y.; Ren, L.-H.; Wang, Z.-C.; Cheng, W.-C. *Green Chem.* **2020,** *22* (4), 1156–1163.
17. Wang, L.; Yu, J.; Duan, Z.; Jin, J.; Zhang, Y. *Org. Biomol. Chem.* **2022,** *20* (33), 6554–6557.
18. Deng, Y.; Lu, S.-C.; Yue, L.-L.; Gong, Y.-L.; Guan, X.-D. *Synth. Commun.* **2022,** *52* (23), 2198–2204.
19. Li, W.; Zhou, P.; Zhao, Q.; Lin, K.; Zhu, T. *Org. Biomol. Chem.* **2023,** *21* (18), 3784–3788.
20. Fernandes, R. A.; Bethi, V. *RSC Adv.* **2014,** *4* (76), 40561–40568.

7 NMR spectra

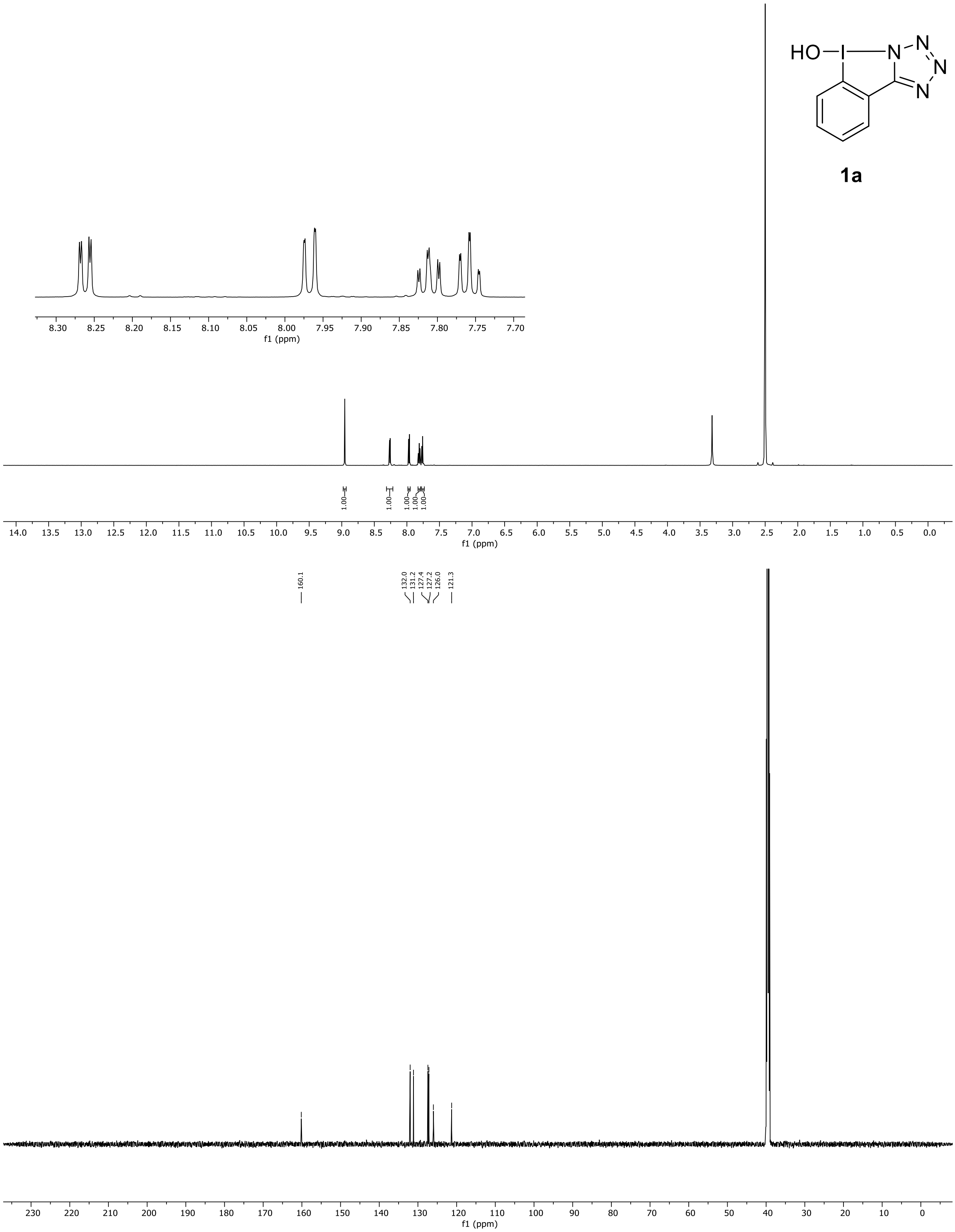

Figure S3: ^{1}H and ^{13}C NMR spectra of **1a** in DMSO-d_6.

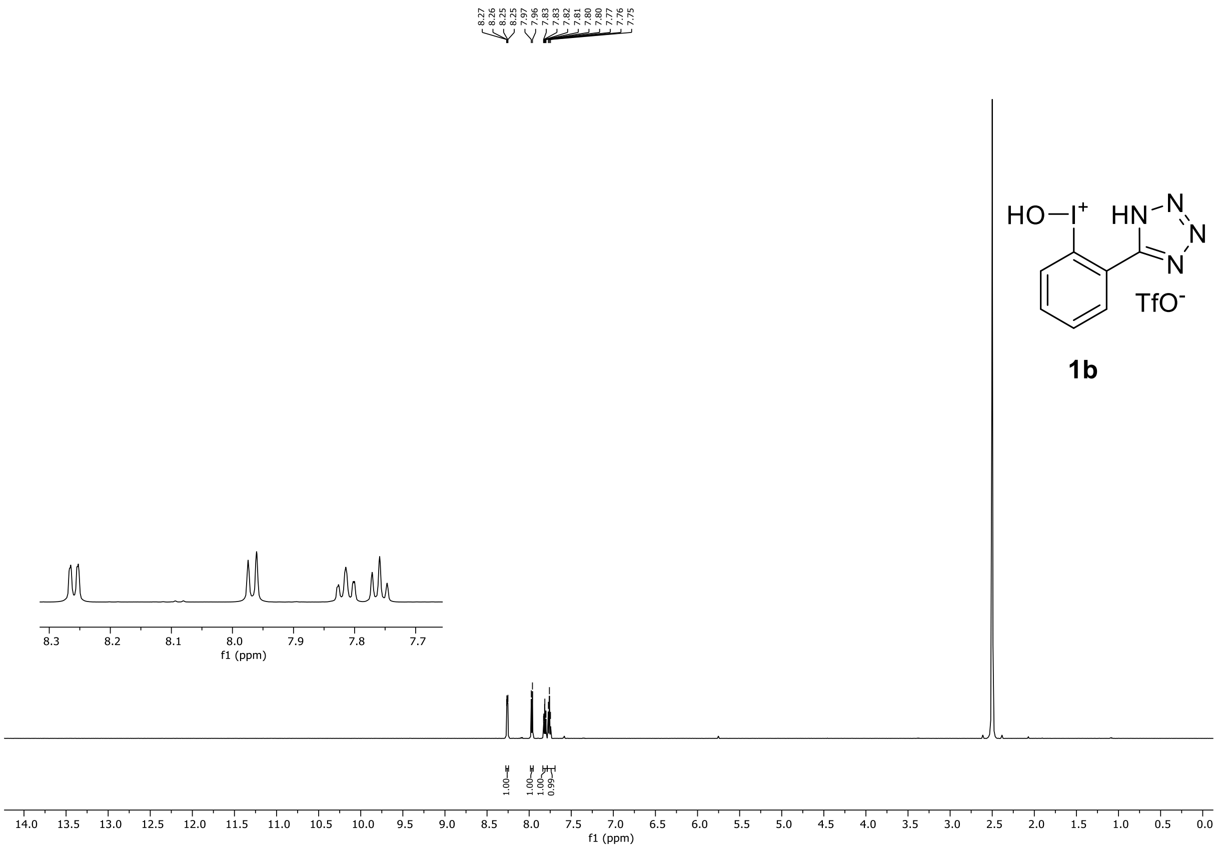

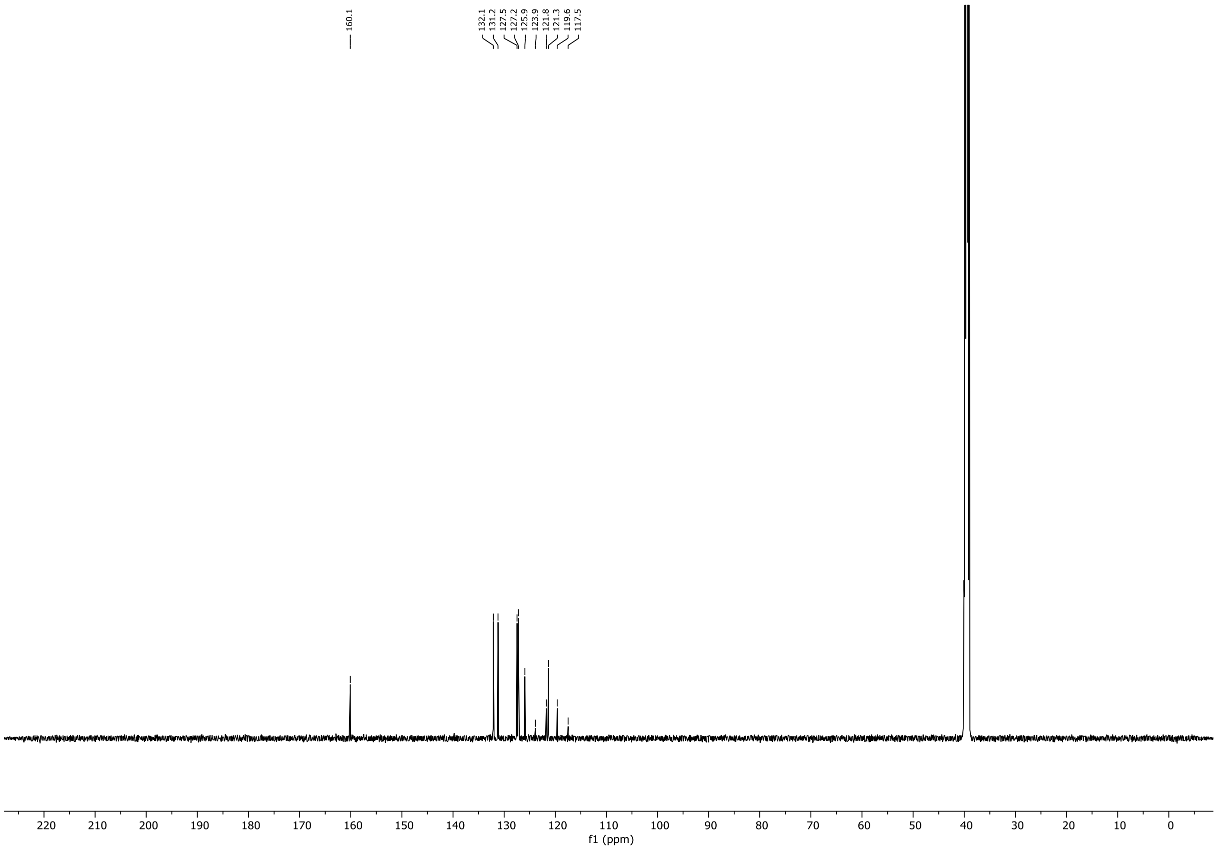

Figure S4: ^{1}H and ^{13}C NMR spectra of **1b** in DMSO-d_6.

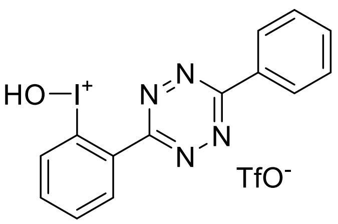
HO—I+
N
N
N
N
TfO-

1c

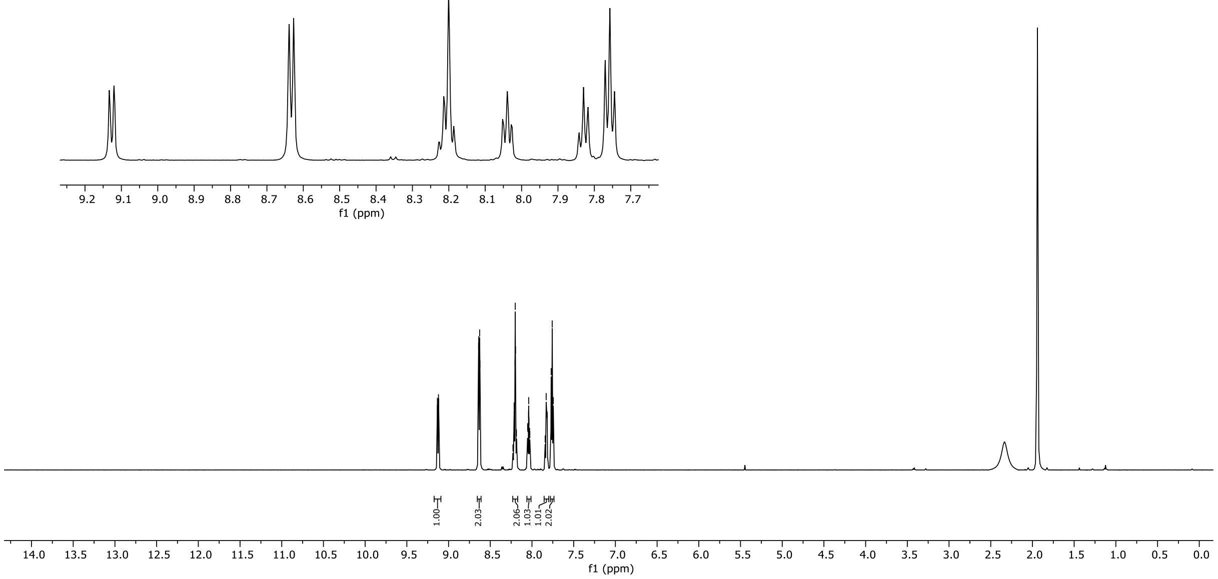
9.13
9.13
9.12
9.12
8.64
8.63
8.62
8.23
8.23
8.22
8.21
8.21
8.20
8.20
8.19
8.18
8.05
8.05
8.04
8.04
8.04
8.03
8.03
7.84
7.83
7.82
7.77
7.76
7.75
7.74
1.00
2.03
2.06
1.03
1.01
2.02
f1 (ppm)
f1 (ppm)

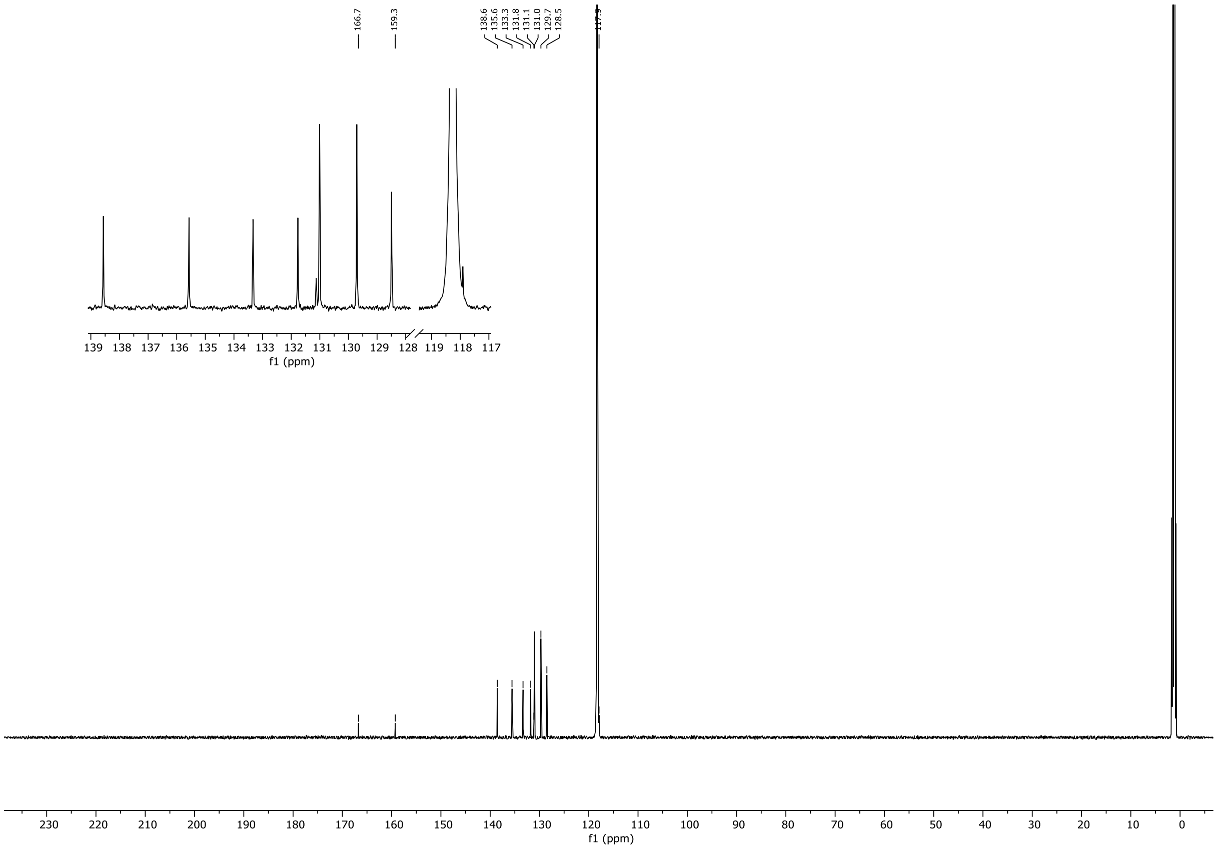
166.7
159.3
138.6
135.6
133.3
131.8
131.1
131.0
129.7
128.5
f1 (ppm)
f1 (ppm)

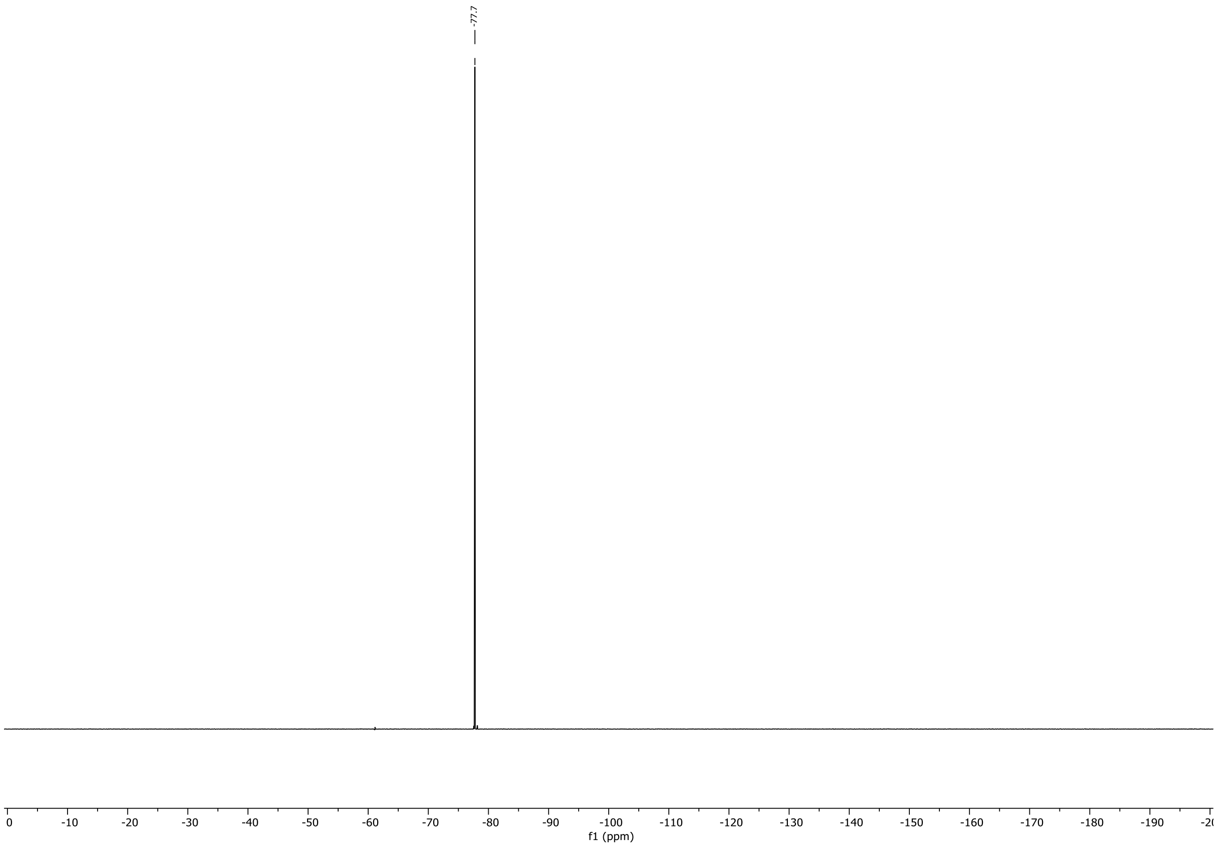

Figure S5: ^{1}H, ^{13}C and ^{19}F NMR spectra of **1c** in CD_3CN.

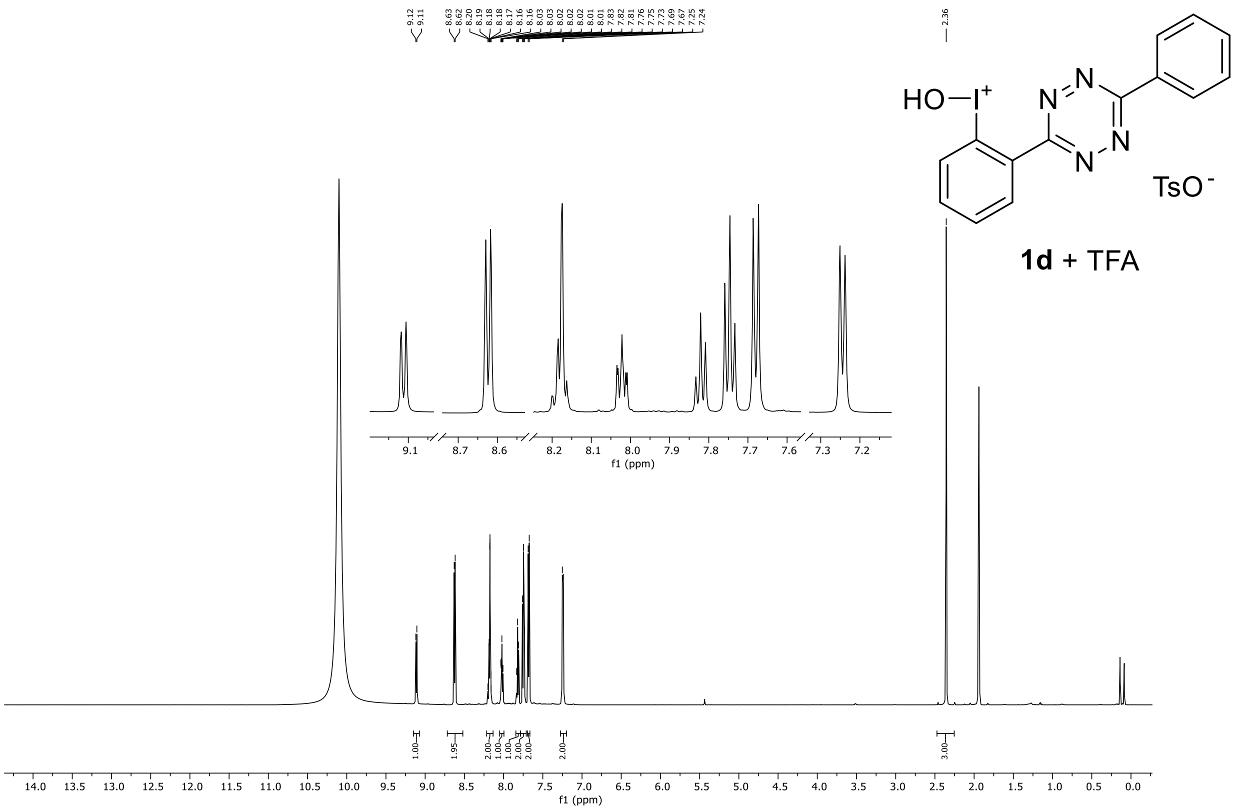

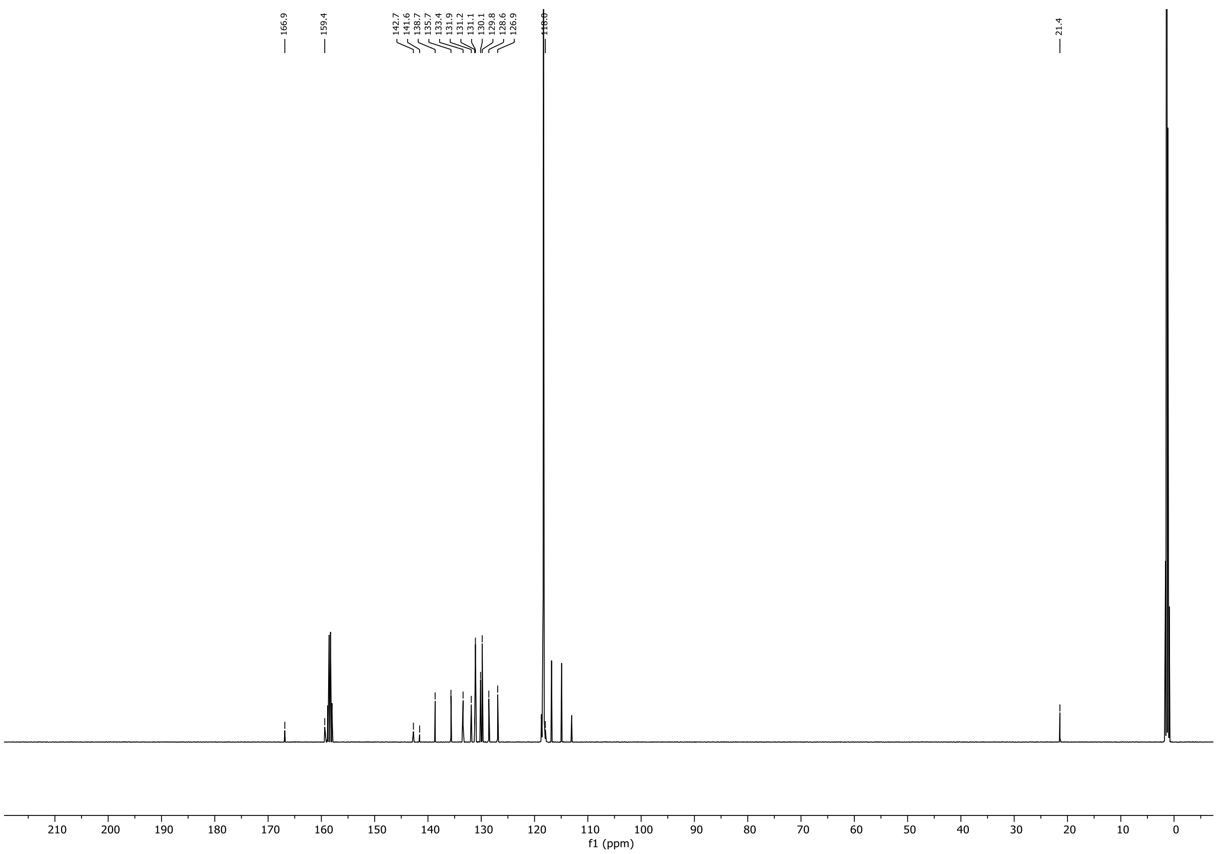

Figure S6: ^{1}H, and ^{13}C and NMR spectra of **1d** in CD_3CN with a few drops of trifluoroacetic acid.

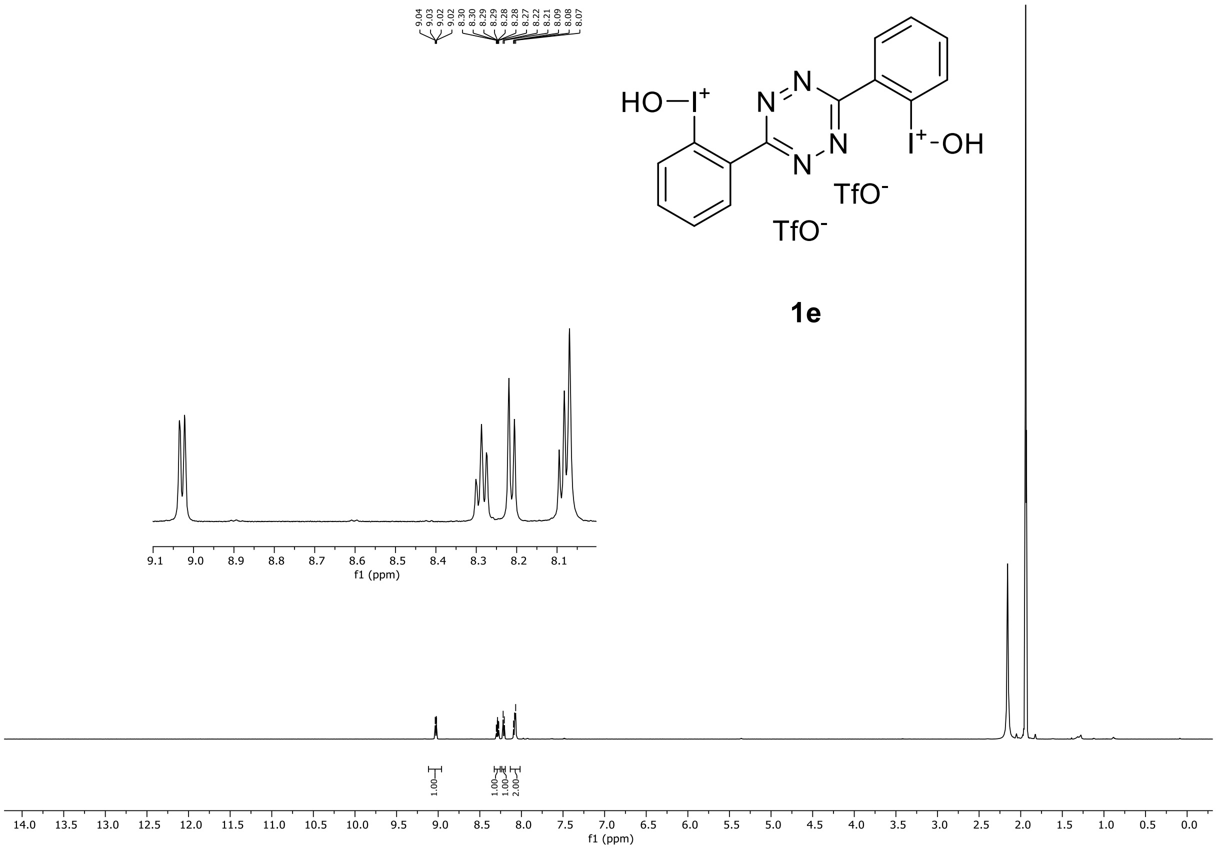
9.04
9.03
9.02
9.02
8.30
8.30
8.29
8.28
8.28
8.27
8.22
8.21
8.09
8.08
8.07
f1 (ppm)
1.00
1.00
1.00
2.00
f1 (ppm)
HO–I+
N
N
N
N
I+-OH
TfO-
TfO-

1e

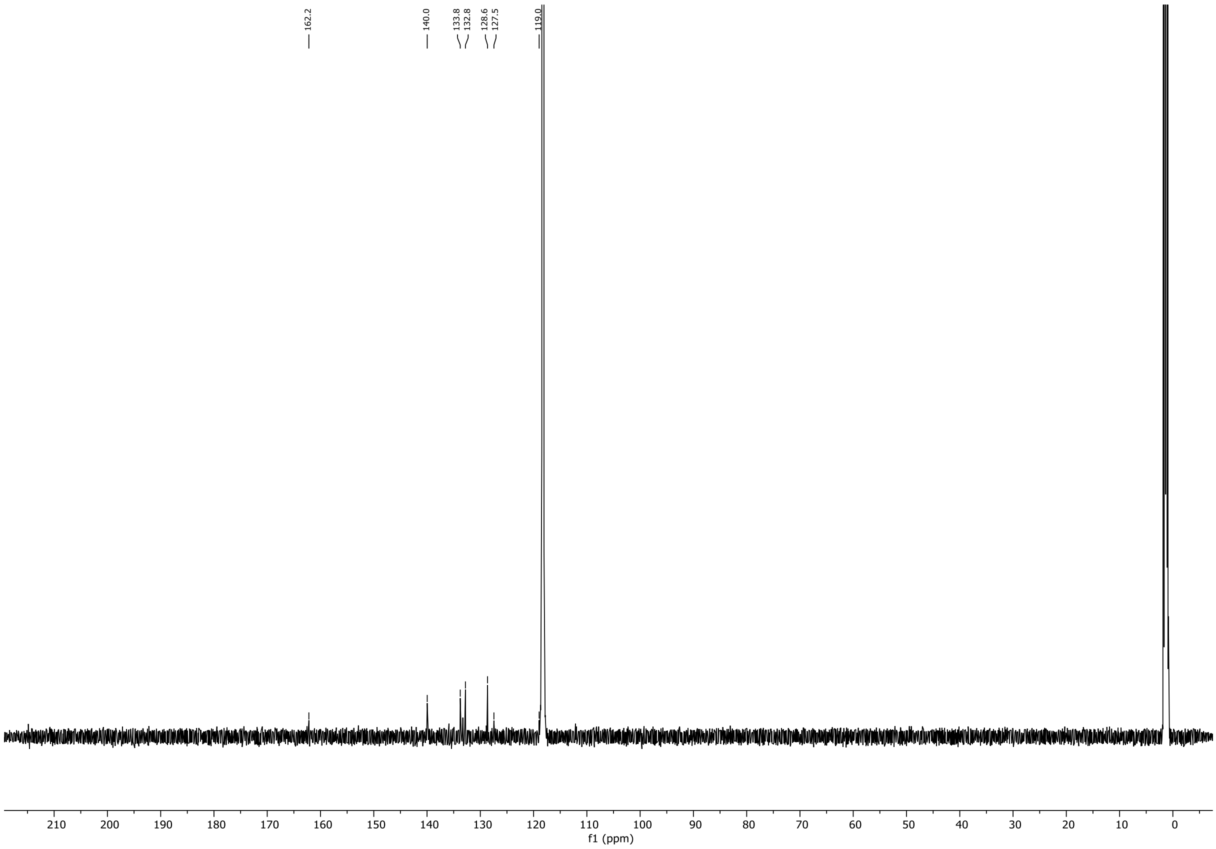
162.2
140.0
133.8
132.8
128.6
127.5
119.0
f1 (ppm)

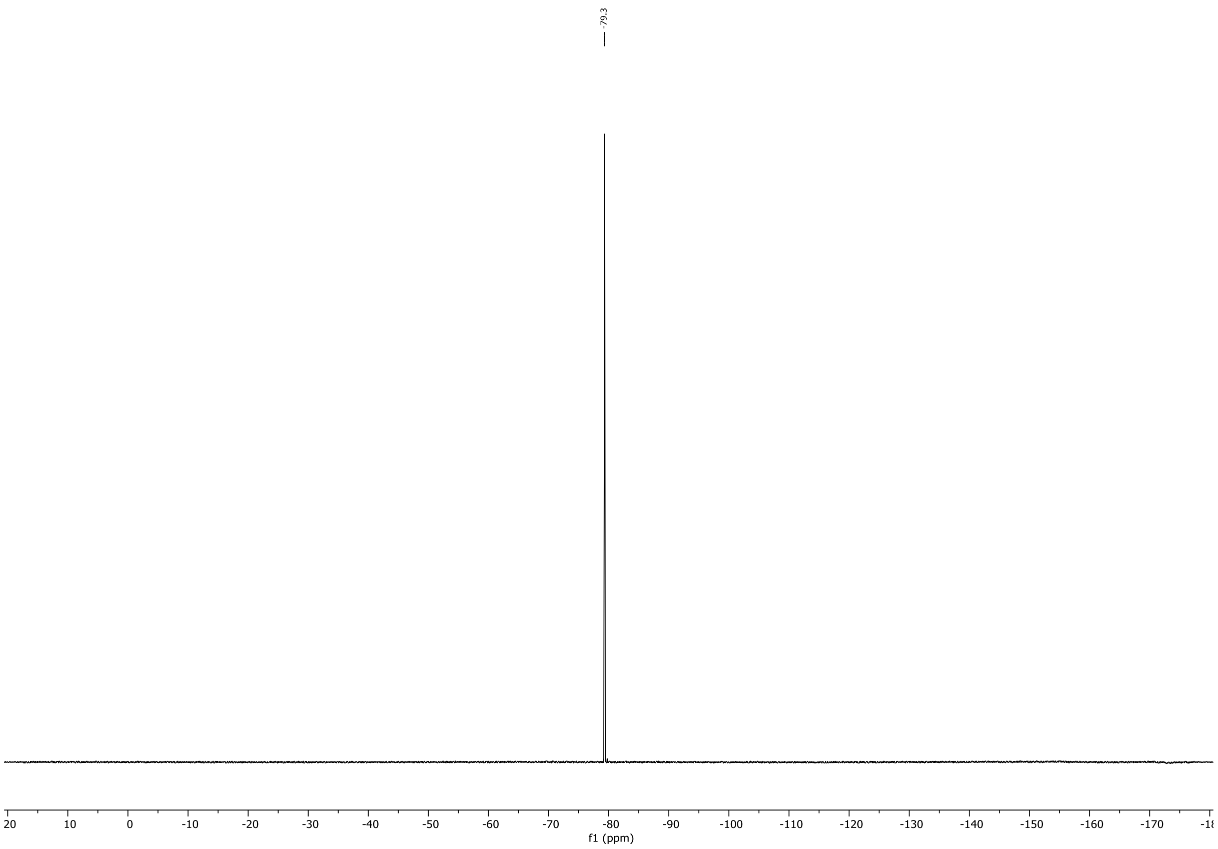

Figure S7: ^{1}H, ^{13}C and ^{19}F NMR spectra of **1e** in CD_3CN.

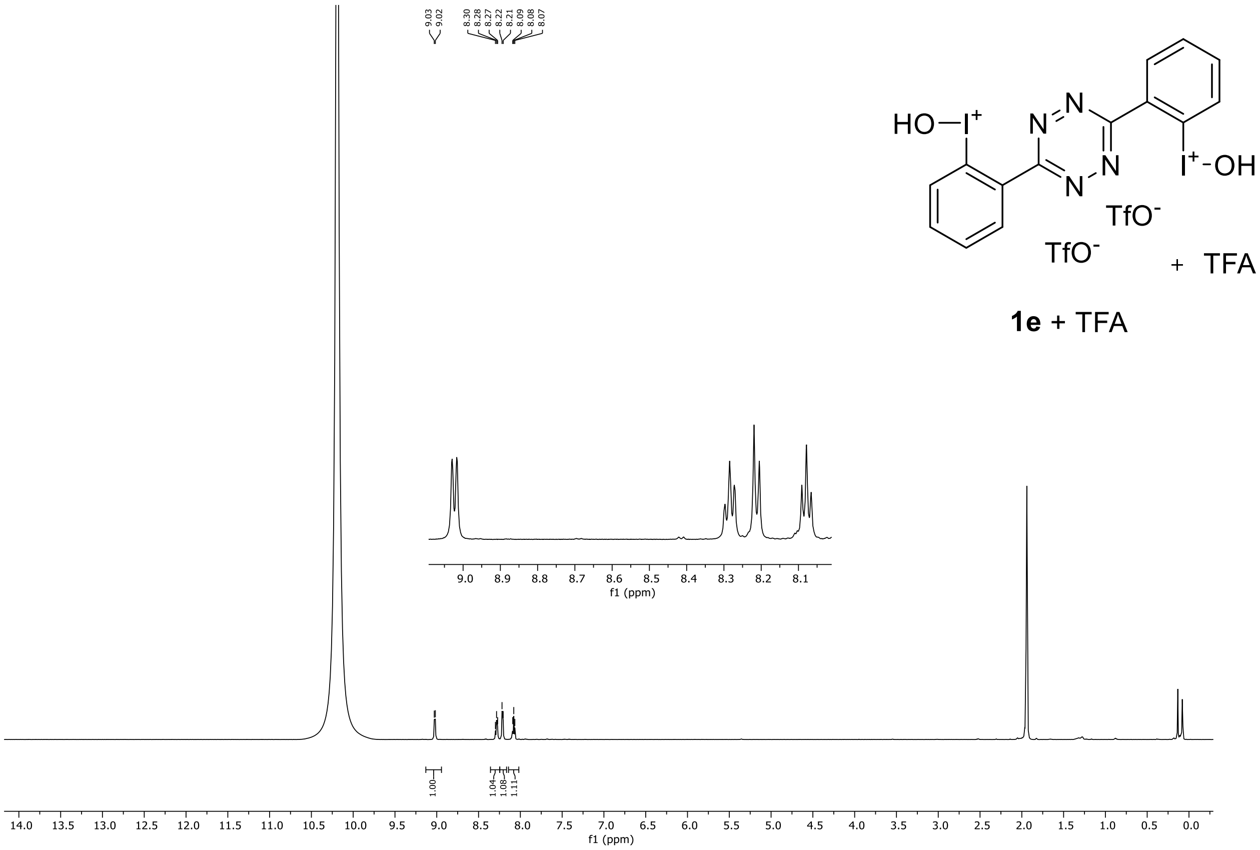

HO—I+
N
N
I+-OH
TfO-
TfO-
+ TFA
1e + TFA
9.03
9.02
8.30
8.28
8.27
8.22
8.21
8.09
8.08
8.07
1.00
1.04
1.08
1.11
f1 (ppm)

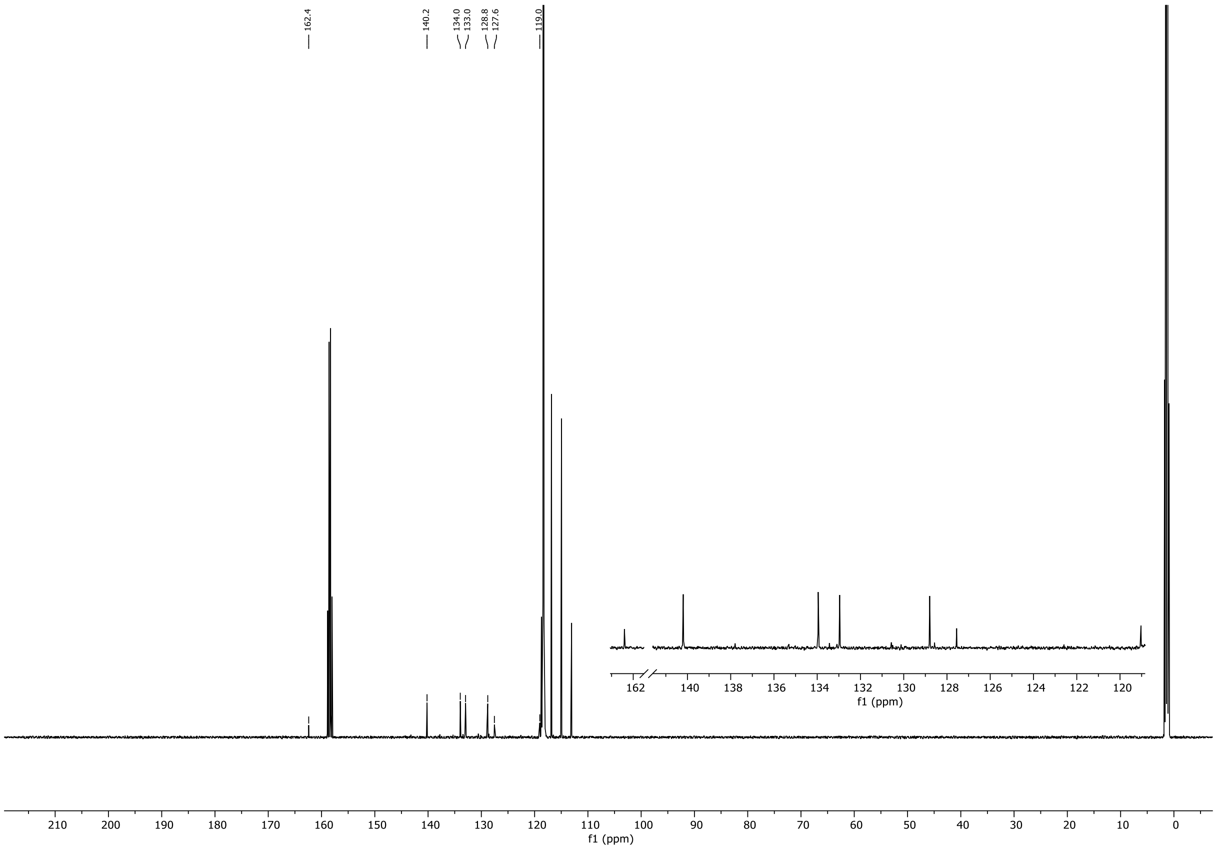

162.4
140.2
134.0
133.0
128.8
127.6
119.0
f1 (ppm)

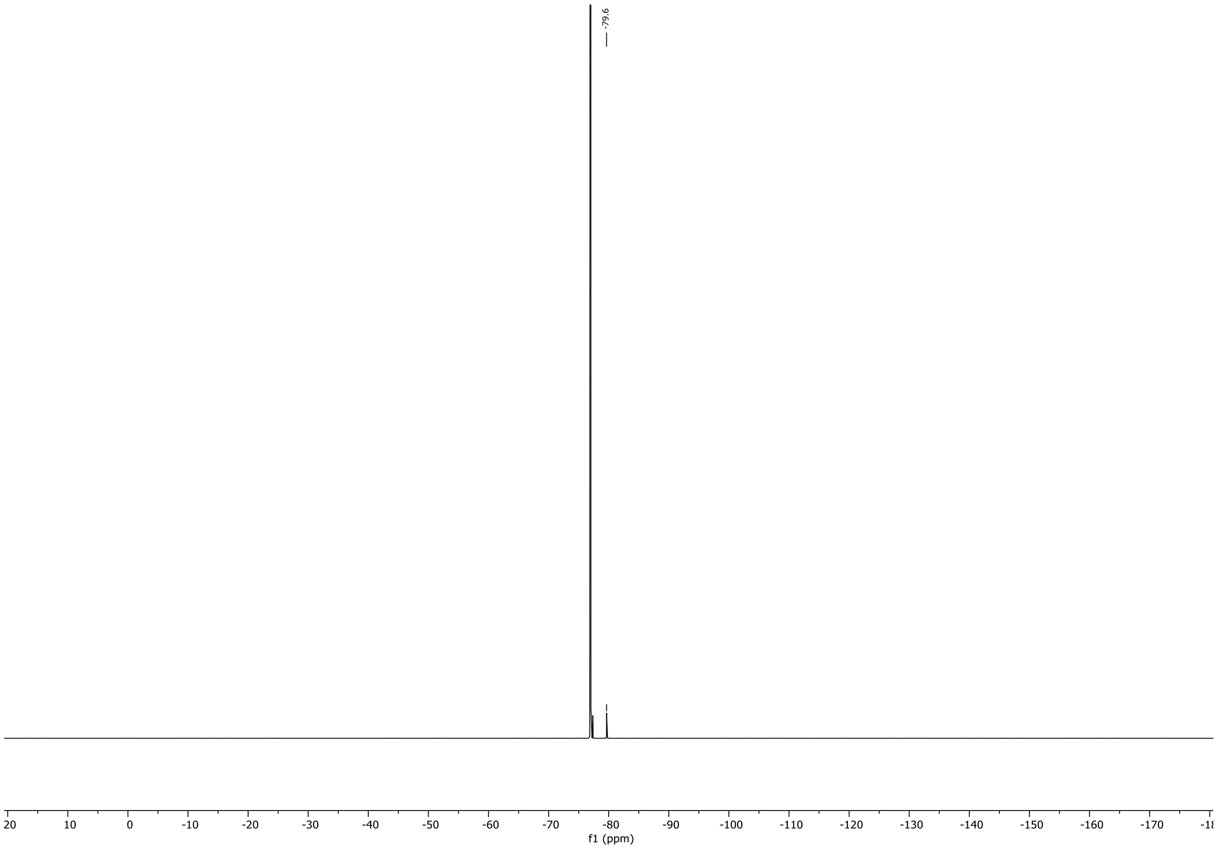

Figure S8: ^{1}H, ^{13}C and ^{19}F NMR spectra of **1e** in CD_3CN with a few drops of trifluoroacetic acid.

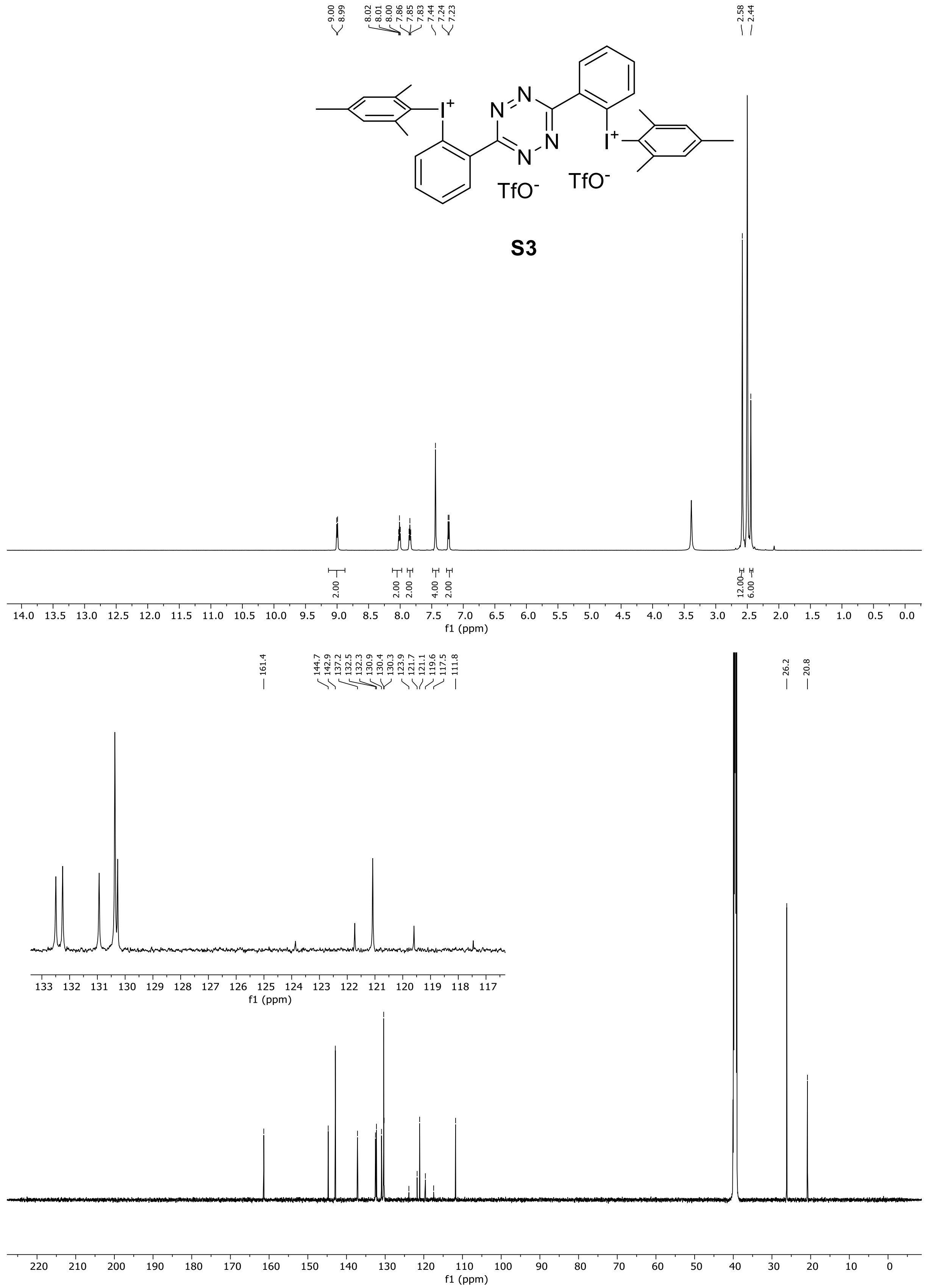

Figure S9: ^{1}H and ^{13}C NMR spectra of **S3** in DMSO-d_6.

8 Crystal structure of 1c

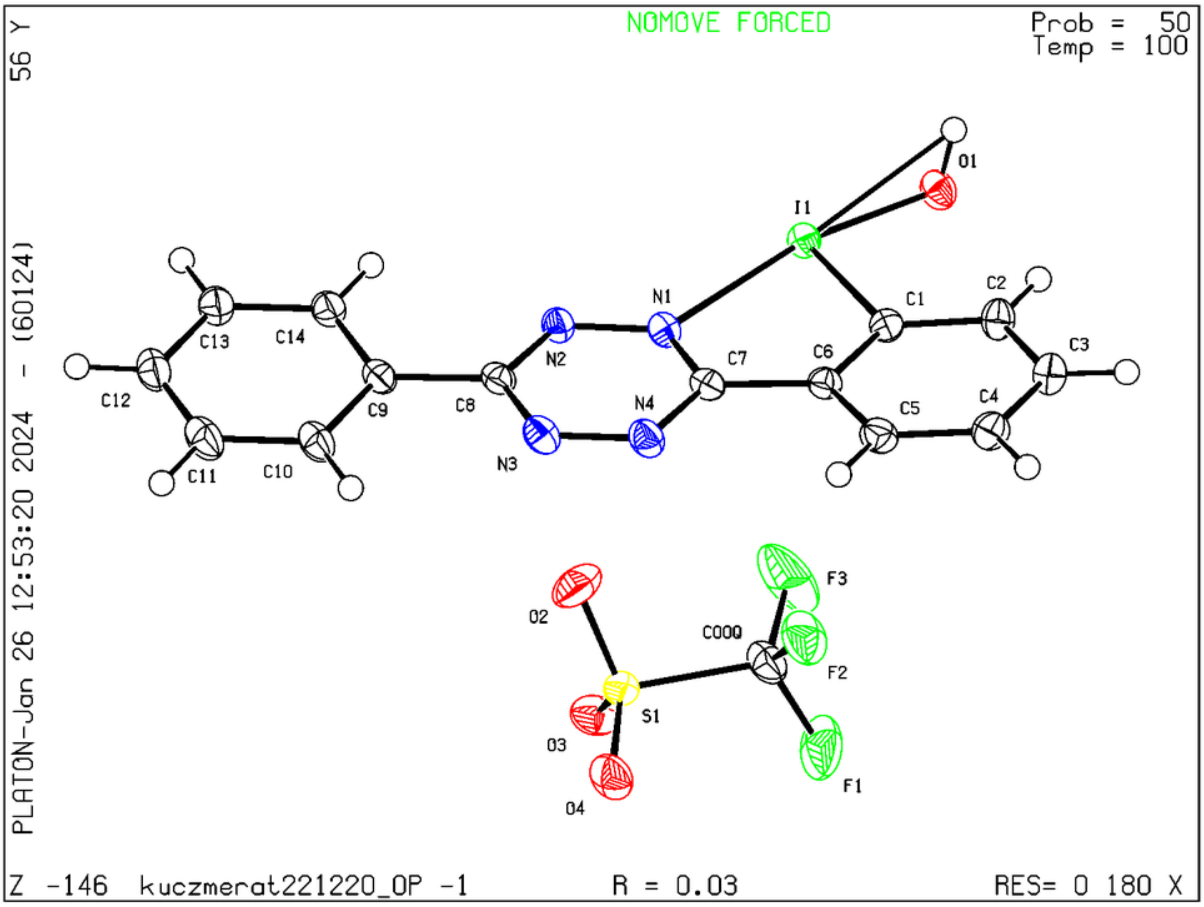

Bond precision: C-C = 0.0030 A Wavelength=0.71073

Cell:	a=8.8057(5)	b=10.2200(6)	c=10.7938(6)
	alpha=75.783(3)	beta=69.696(3)	gamma=82.323(3)

Temperature: 100 K

	Calculated	Reported
Volume	881.87(9)	881.87(9)
Space group	P -1	P -1
Hall group	-P 1	-P 1
Moiety formula	C14 H10 I N4 O, C F3 O3 S	C14 H10 I N4 O, C F3 O3 S
Sum formula	C15 H10 F3 I N4 O4 S	C15 H10 F3 I N4 O4 S
Mr	526.23	526.23
Dx,g cm-3	1.982	1.982
Z	2	2
Mu (mm-1)	1.997	1.997
F000	512.0	512.0
F000'	511.58	
h,k,lmax	14,17,18	14,17,18
Nref	8629	8579
Tmin,Tmax	0.622,0.774	0.601,0.747
Tmin'	0.609	

Correction method= # Reported T Limits: Tmin=0.601 Tmax=0.747 AbsCorr = MULTI-SCAN

Data completeness= 0.994 Theta(max)= 36.483

R(reflections)= 0.0307(7384) wR2(reflections)= 0.0691(8579)

S = 1.024 Npar= 257
